# Nucleolin Aptamer N6L Reprograms the Translational Machinery and Acts Synergistically with mTORi to Inhibit Pancreatic Cancer Proliferation

**DOI:** 10.3390/cancers13194957

**Published:** 2021-10-01

**Authors:** Mounira Chalabi-Dchar, Elisabeth Cruz, Hichem C. Mertani, Jean-Jacques Diaz, José Courty, Ilaria Cascone, Philippe Bouvet

**Affiliations:** 1 Centre de Recherche en Cancérologie de Lyon, Université de Lyon 1, Inserm U1052, CNRS UMR5286 Centre Léon Bérard, CEDEX 08, F-69373 Lyon, France; Mounira.CHALABI@lyon.unicancer.fr (M.C.-D.); Elizabeth.CRUZGOMEZ@lyon.unicancer.fr (E.C.); hichem.mertani@lyon.unicancer.fr (H.C.M.); JeanJacques.DIAZ@lyon.unicancer.fr (J.-J.D.); 2 INSERM, Institut Mondor de Recherche Biomédicale (IMRB), Université Paris-Est Créteil, F-94010 Créteil, France; courty@u-pec.fr (J.C.); ilaria.cascone@u-pec.fr (I.C.); 3 Ecole Normale Supérieure de Lyon, Université de Lyon 1, F-69007 Lyon, France

**Keywords:** nucleolin, pancreatic cancer, translation, mTOR inhibitor, combotherapy

## Abstract

**Simple Summary:**

Pancreatic cancer is an aggressive disease characterized by its invasiveness, rapid progression, and resistance to conventional therapy. There is a need to identify new molecules to improve current therapies. The aim of this study was to analyze how the pancreatic cancer cells react to the treatment with an inhibitor of nucleolin, N6L. To this end, we analyzed how the translation was affected in the cells during the treatment. We discovered that in response to N6L, a signaling pathway called the mTOR pathway was activated and was involved in the activation of translation of a subset of mRNA that could be involved in the resistance of the cells to the treatment. Indeed, we showed that the combined action of inhibitors of the mTOR pathway with N6L synergistically inhibited the cancer cells’ proliferation. We propose that this new combination of molecules could be a novel therapeutic option for pancreatic cancer.

**Abstract:**

We previously showed that N6L, a pseudopeptide that targets nucleolin, impairs pancreatic ductal adenocarcinoma (PDAC) growth and normalizes tumor vessels in animal models. In this study, we analyzed the translatome of PDAC cells treated with N6L to identify the pathways that were either repressed or activated. We observed a strong decrease in global protein synthesis. However, about 6% of the mRNAs were enriched in the polysomes. We identified a 5′TOP motif in many of these mRNAs and demonstrated that a chimeric RNA bearing a 5‘TOP motif was up-regulated by N6L. We demonstrated that N6L activates the mTOR pathway, which is required for the translation of these mRNAs. An inhibitory synergistic effect in PDAC cell lines, including patient-derived xenografts and tumor-derived organoids, was observed when N6L was combined with mTOR inhibitors. In conclusion, N6L reduces pancreatic cells proliferation, which then undergoes translational reprogramming through activation of the mTOR pathway. N6L and mTOR inhibitors act synergistically to inhibit the proliferation of PDAC and human PDX cell lines. This combotherapy of N6L and mTOR inhibitors could constitute a promising alternative to treat pancreatic cancer.

## 1. Introduction

Pancreatic ductal adenocarcinoma (PDAC) is a highly aggressive disease, for which mortality closely parallels incidence with a 5-year survival rate of less than 6% [1]. It is predicted to be the second leading cause of cancer-related death by 2030 [2]. The biology of PDAC contributes to early recurrence and metastasis, resistance to chemotherapy and radiotherapy, because of its complexity at the genomic, epigenetic, and metabolic levels, with multiple activated biological pathways and crosstalk. Although there are four major driver genes identified in PDAC, including one oncogene, KRAS, and three tumor suppressor genes such as *CDKN2A* (encoding p16), *TP53* and *SMAD4,* none of them has currently provided clues to treat the disease.

Currently, a cure can only be achieved through resection. However, for many patients with advanced disease, surgery is not the option. However, if they have suitable performance status, some of these patients could receive FOLFIRINOX (folinic acid or leucovorin, fluorouracil, irinotecan, and oxaliplatin) and gemcitabine/NAB-paclitaxel (nanoparticle albumin-bound paclitaxel) as neoadjuvant treatments [3,4]. Indeed, therapeutic options are limited, and discovering effective drug therapies for affected patients is of paramount importance.

Nucleolin (NCL), an RNA- and protein-binding multifunctional protein, has become the focus of interest in the cancer biology field in recent years for several reasons. First, its expression seems to correlate with the proliferation rate of the cells with overexpression in several cancer types, including hepatocellular carcinoma [5], acute myeloid leukemia [6], non-small-cell lung cancer [7], gastric cancer [8], and PDAC [9]. Second, in addition to its nucleolar, nucleoplasmic, and cytoplasmic localization, it is present on the surface of many cell types, and this extracellular form of NCL is a hallmark of proliferative and cancer cells [10,11,12,13].

Indeed, NCL is a key protein for the regulation of several processes required for the proliferation and division of the cells. In particular, it plays an important role in the coordination of cell metabolism with cell division [14] and in the biogenesis of ribosomes, which are required to sustain the higher level of translation in cells that have a high proliferation rate. The high expression level of NCL in cancer cells is related to a high level of protein synthesis.

Several molecules were developed to target NCL on the cell surface of cancer cells. Among them, the pseudopeptides HB19 and N6L were shown to interact with NCL and to inhibit the proliferation of cancer cells [15,16]. In addition, N6L, as well as an NCL-blocking antibody, impairs both in vitro and in vivo angiogenesis by targeting endothelial cells and tumor vessels [17,18]. Many studies reported the antitumor activities of N6L in several cancer types including, breast cancer [17], glioblastoma [19], non-small cell lung carcinoma [20], and PDAC [9,21].

Interestingly in PDAC, NCL targeting by N6L blocks both tumor progression and normalizes tumor vasculature, improving the delivery and efficacy of chemotherapeutic drugs [9].

In the present study, in order to better understand how the NCL antagonist, N6L, inhibits cancer cell growth, we carried out a translatome analysis in mPDAC cell lines. Using this approach, we showed that N6L induces translational reprogramming through the activation of the mTOR pathway during the treatment. Interestingly, we showed that N6L and mTOR inhibitors act synergistically to inhibit the proliferation of several PDAC and human PDX cell lines.

## 2. Materials and Methods 

### 2.1. Materials and Cell Lines

The nucleolin antagonist N6L (NUCANT^®^) was obtained from the UREKA Pharma company (Pessac, France). The mTOR inhibitors, rapamycin (Sirolimus from Pfizer^®^(New York, NY, USA)) and INK128 (Sapanisertib, TAK128, or MLN128 from Takeda oncology^®^(Cambridge, MA, USA) were purchased from Selleckchem laboratories (PA, USA), and stock solutions were made in DMSO.

PDAC cell lines, MiaPaca-2, Panc-1, mPDAC were maintained in DMEM medium, and HeLa cells were maintained in αMEM medium containing Glutamax (PAA) 1% non-essential amino acids, supplemented with 10% FBS and 1% Penicillin/Streptomycin. PDAC PDX cell lines were purchased from CTIBIOTECH(Lyon, France) and maintained in CTIM Cancer2 medium (Lyon, France). All cells were incubated at 37 °C in a humidified incubator with 5% CO_2_. Routine mycoplasma testing was performed by MycoAlert Mycoplasma Detection Kit (catalog no. LT07-118).

### 2.2. Spheroid and Organoid Formation

For the spheroid assay, PDAC cell lines were cultured in 96-well ultra-low attachment (ULA) plate and allowed to form a spheroid overnight. The next day, PDAC spheroids were treated or not with different concentrations of N6L alone or in combination with mTORi, RAPA, and INK128 in 4 replicates. Spheroid growth was monitored by IncuCyte ZOOM^®^ (Michigan, USA) live-cell imaging. Over 96 h, the spheroid area was calculated using ImageJ software, and data were normalized to control.

For organoid formation, mPDAC cells (1 × 10^3^ cells/mouse in 50 µL PBS) were orthotopically injected into the pancreas of FVB/n syngeneic mice, as previously described [9]. Mice were sacrificed after 4 weeks, and tumor-derived organoids were established by following Tuveson’s laboratory protocol [22]. Briefly, tumors were digested with 0.012% (*w*/*v*) collagenase XI (Sigma (Burlington, MA, USA) and 0.012% (*w*/*v*) dispase (Gibco (Waltham, MA, USA) in DMEM media containing 1% FBS, and the resulting cell suspension was incorporated into growth factor-reduced matrigel (Corning (Corning, NY, USA)) to obtain a 3D culture. All in vivo experiments were carried out with the approval of the appropriate ethical committee and under conditions established by the European Union.

### 2.3. Viability Assay (MTS) and Apoptosis Analysis

Cell viability was assessed by MTS assay (CellTiter 96 AQueous MTS Reagent, Promega (Madison, WI, USA). Briefly, cell medium was supplemented with MTS reagent, incubated for 2 h, and then the absorbance at 450 nm was recorded on TECAN microplate reader (Sunrise^®^ (Basel, Switzerland)). Viability for treated cells was normalized to non-treated cells on the same plate.

Apoptosis analysis was performed on the IncuCyte ZOOM^®^ live-imaging with Caspase-3/7 Green Reagent for Apoptosis (Essen BioScience (Ann Arbor, MI, USA)). Cells were treated or not with increasing concentrations of N6L in 96-well plates in triplicate, and Caspase-3/7 reagent was added to the cells. IncuCyte ZOOM^®^ live-imaging software was used to count the fluorescent object numbers per mm^3^ in each well, which reflects the caspase 3/7 activation. Staurosporine (1 µM) was used as a positive control for inducing apoptosis.

### 2.4. Analysis of Synergy/Antagonism from Combination Studies

The effects of nucleolin antagonist (N6L) combined with mTOR inhibitors (mTORi) on PDAC cell growth were analyzed using the freely Combenefit software from CRUCK Cambridge Institut [23], which enables to determine synergy/antagonism using the published Loewe model and attributes a positive score indicates synergy, a score of 0 for additive effect, and a negative score for antagonism. The “3D surface”, “contour”, and “matrix” views were selected as graphical outputs for the synergy distribution and are represented in the Results/Figures section.

### 2.5. Dual-Luciferase Reporter Assay for 5′TOP Constructs

NCL-depleted cells or N6L-treated cells or not were seeded in 96-well plates over 24 h. For the 5′TOP activity assay, cells were co-transfected with a monocistronic reporter containing 5′TOP motif of *Rps3* or *Rpl36* genes (GenScript (Piscataway, NJ, USA) and pRL construct constitutively expressing the *Renilla* luciferase gene as an internal control for transfection efficiency. Luciferase assays were performed 2 h after transfection with the reporter plasmids using X-tremeGENE 9 reagent (Roche (Basel, Switzerland). Dual-luciferase assays were performed using the Dual-Glo luciferase reagent (Promega (Madison, WI, USA) according to the manufacturer’s instructions and a Tecan M1000 plate reader. The relative luciferase activity was calculated by *firefly*-luciferase activity/*Renilla* luciferase activity.

### 2.6. SUnSET for Global Protein Synthesis, Western Blots

Global protein synthesis analysis by puromycylation followed by puromycin detection was performed [24]. Briefly, puromycin (1 µg/mL) was added to the N6L-treated or non-treated cell medium for 2 h at 37 °C. Cells were harvested, lysed in Laemmli buffer, and loaded onto a polyacrylamide gel. Puromycin incorporation was detected by Western blot on whole-cell protein extracts.

Twenty micrograms of total protein lysates were run on a 10–15% SDS polyacrylamide gel and transferred onto a nitrocellulose membrane. The membrane was blocked with 3% nonfat milk in TBST. The antibodies (listed in Table S2) were incubated for 1 h 30 min in 3% milk-TBST. Proteins were detected by chemiluminescence using an anti-rabbit or anti-mouse HRP-conjugated antibody and ECL substrate (Covalab (Bron France). Images were collected on a ChemiDoc XRS+ (Bio-Rad (Hercules, CA, USA), and the signal was analyzed using the Bio-Rad ImageLab software. The original western blots can be found in Figure S6.

### 2.7. Polysome Profiling and RNA Extraction and Sequencing

Cells were cultured in 15 cm dishes and were treated or not for 48 with 50 µM N6L. Before cell lysis, cells were incubated with emetine then washed with cold PBS as previously published by our lab [25]. For cell fractionation, 10 × 10^6^ cells were resuspended in lysis hypotonic buffer for 10 min at 4 °C. Cell lysates were successively centrifuged at 700 g and 1200 g to eliminate the nuclei and the mitochondria, respectively. Cytoplasmic lysates (1–2 mg of proteins) were loaded onto 15–47% sucrose density gradients and centrifuged at 217,000 g for 2 h at 4 °C. Gradients were fractionated into 19 fractions, and the OD at 254 nm was continuously recorded using an ISCO fractionator (Teledyne ISCO (Lincoln, NE, USA). RNA from cytosolic and polysomal pooled fractions was extracted and purified using TRIzol reagent (Invitrogen, (Waltham, MA, USA)).

High-throughput RNA sequencing (Illumina (San Diego, CA, USA) was performed at the Ecole Normale Superieure genomic core facility (Paris, France) as previously published [26], and the reads were analyzed using STAR (version 2.5.2b) [27], samtools [28], HTSeq-count 0.5.3 [29]. The RNASeq gene expression data and raw fastq files are available on the GEO repository (www.ncbi.nlm.nih.gov/geo/, accessed on 15 September 2021) under accession number: GSE184857. Statistical treatments and differential analyses were also performed using DESeq2 1.8.1 [30].

### 2.8. Statistics

Statistical analyses were performed by using GraphPad Prism software (version 6). Bars represent mean ± SEM (*n* ≥ 3). For two-group comparisons, we analyzed the data using a two-tailed Student *t*-test. For multiple group comparisons, 1-way ANOVA rank with Dunn method was used (* *p* < 0.05, ** *p* < 0.01, *** *p* < 0.005, **** *p* < 0.001) and n.s. for not statistically significant.

## 3. Results

### 3.1. NCL Targeting by N6L Impairs Protein Synthesis and Induces a Translational Reprogramming in mPDAC Cells

**Figure 1:**
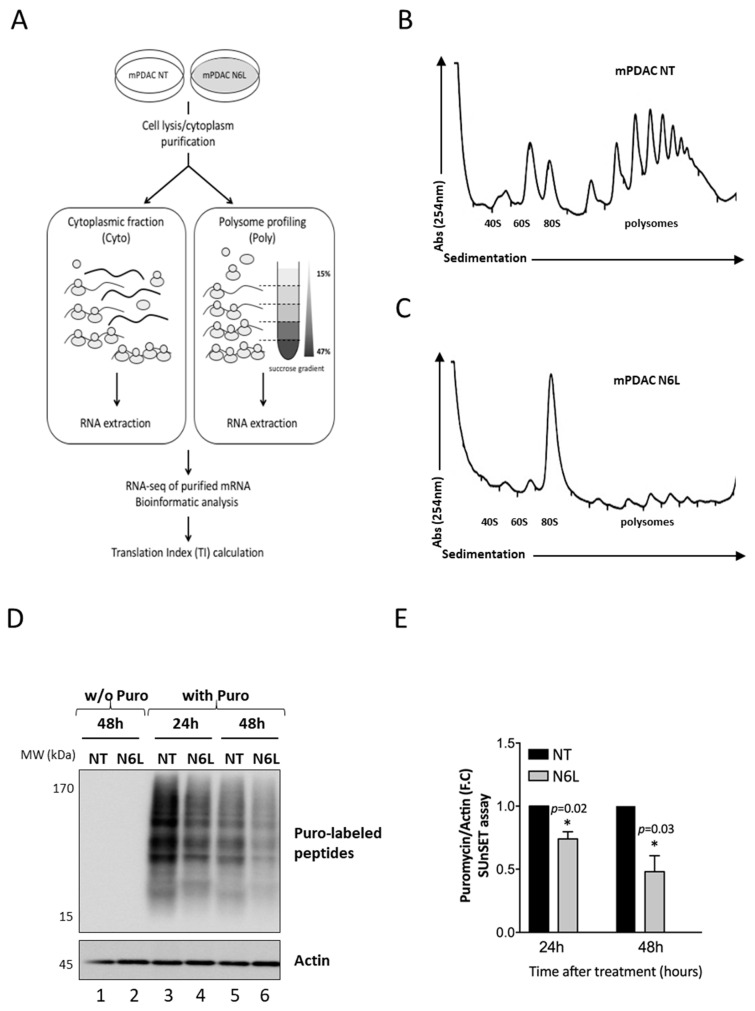
NCL targeting by N6L impacts mPDAC cell translation. (**A**)—Experimental design of translatome analysis procedure. mPDAC cells were treated (T) or not (NT) with 50 µM of N6L for 48 h. Cytosolic lysates from cells were sedimented on a sucrose gradient (15–47%). Following ultracentrifugation, 40S and 60S ribosome subunits, the 80S monosomes, and polysomes are separated. RNAs were extracted from polysome pooled fractions (mRNA-associated polysomes) and cytoplasmic fractions (cytoplasmic mRNA). The RNA quality and integrity were verified using a Bioanalyser 2100 before sequencing and bioinformatics analysis. (**B**,**C**)—Polysome profiles of mPDAC cells non-treated (mPDAC NT) or treated (mPDAC T) with N6L. Curves show absorbance at 254 nm as a function of sedimentation. (**D**,**E**)—mPDAC cells, treated or not with N6L, were incubated with puromycin for the measurement of overall protein synthesis by the SUnSET method and then subjected to Western blotting (**D**). Protein synthesis rates were quantified by measuring the signal intensity in each lane and normalizing the values to that of the control lane (**E**). w/o Puro, without puromycin. Data represented are the means ± SD of at least three experiments. * *p* < 0.05 and n.s. for not significant, as determined by Student’s *t*-test on final data point.

mRNA translation is a central cellular process that regulates growth and metabolism [31]. To evaluate the impact of N6L treatment on mPDAC translatome, we performed polysome profiling analysis on cells treated or not with N6L for 48 h (Figure 1).

Time-course analysis of mPDAC treated with different concentrations of N6L shows that at 30–50 µM, a strong reduction in cell proliferation is observed (Figure S1A) while metabolic activity, which reflected cell viability, assessed by MTS test, is reduced by 56% (Figure S1B).

To evaluate the impact of N6L treatment on mPDAC translatome, treated and non-treated mPDAC cells were harvested, and RNAs were extracted from an aliquot of the cytoplasmic fractions while the remaining fraction was analyzed through sucrose gradient to perform polysome profiling. RNAs were then extracted from the pooled polysomal fractions. The extracted RNAs (from cytoplasmic and polysomal pooled fractions) were submitted to deep sequencing (workflow shown in Figure 1A).

The polysome profile of mPDAC non-treated cells (Figure 1B) shows the classical profile of ribosome distribution. In contrast, when cells were treated with N6L (Figure 1C), a strong decrease in polysome peaks is observed with a reduced level of 40S and 60S subunits and an important increase in the 80S peak in comparison to non-treated cells.

In order to evaluate the ability of mPDAC cells to synthesize proteins following N6L treatment, we used the surface sensing of translation (SUnSET) technique [24], which is based on the incorporation of puromycin to newly synthesized proteins and its detection with anti-puromycin antibodies (Figure 1). As shown in fig1.D-E, mPDAC-treated cells displayed a significant decrease (26%) in the amount of puromycin-labeled peptides, compared to non-treated mPDAC as soon as after 24 h of N6L treatment (lane 4) with a maximum decrease of 52% after 48 h (lane 6) (Figure 1D,E).

These results indicated a decrease in protein synthesis in N6L-treated cells and agree with the decrease in the mPDAC translation capacity upon N6L treatment.

**Figure 2:**
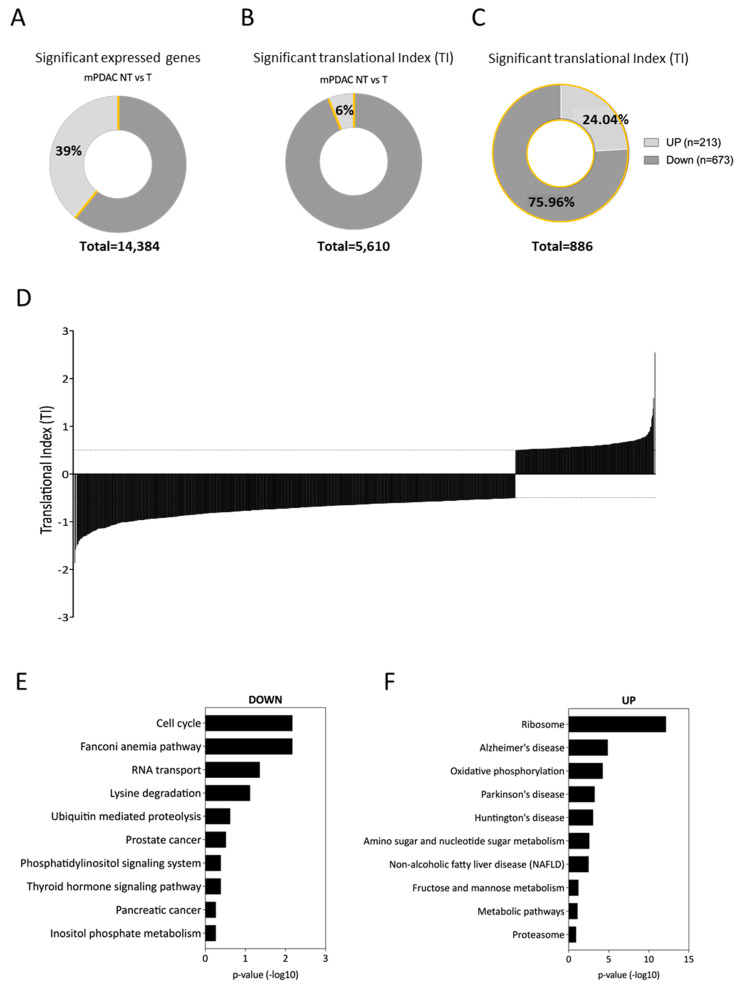
Bioinformatics analysis of mPDAC cell translatome upon N6L treatment. **A**—Percentage of translationally deregulated mRNAs in response to N6L treatment. Among the 14,384 blasted genes, 39% (5610) were significantly expressed in both NT and T conditions (**A**). About 6% (886) of these 5610 genes were significantly deregulated at translational levels (TI cut-off = 1.5, *p* < 0.05) (**B**). Among these 886 translationally deregulated genes, 24.04% were up-regulated, and 75.96% were down-regulated (**C**). Distribution of the Translational Index (TI) (TI = X1/X2), with X1 and X2 expressing the ratio between polysomal-to-cytoplasmic fraction in mPDAC-treated and non-treated cells, respectively (**D**) (complete gene list in Table S1). (**E**,**F**)—Ontology analysis was determined using the functional annotation clustering tools from EnrichR software. Translationally down-regulated genes are involved in the cell cycle, Fanconi anemia and RNA transport (**E**). While translationally up-regulated genes are mainly involved in ribosome/translation and metabolism (**F**).

To determine how the translatome was affected by N6L treatment, results from the deep sequencing of cytoplasmic and polysomal fractions were analyzed (Figure 2). The RNA-sequencing analysis using the package DeSeq2 showed that among 14,384 mRNAs present in mPDAC, 39% (5610) were significantly expressed in both non-treated and N6L-treated cells (*p*-adj < 0.05) (Figure 2A).

These significantly expressed genes in non-treated and treated mPDAC cells were used to identify *bona fide* alterations in translational efficiency in response to N6L by calculating the translational index (TI) (Figure 2D). TI reflects the translation efficiency for each expressed mRNA by measuring the percentage of mRNA engaged in the polysomal (poly) fractions to a total amount of this mRNA found in the cytoplasmic (cyto) fractions and comparing these percentages between treated (T) and non-treated (NT) mPDAC cells (TI = X1/X2, with X1 and X2 representing the ratio between polysomal-to-cytoplasmic fraction in mPDAC-treated and non-treated cells, respectively).

By applying a TI cut-off = 1.5 and *p*-adj < 0.05, we identified 886 genes whose TI was significantly changed in response to N6L over 48 h (Figure 2C), indicating that N6L treatment altered the translation efficiency of 886 mRNAs out of the 14,384 analyzed. The range of TI varying from −2 to +2.5 (Figure 2D). The translation of 673 (75.96%) of these mRNAs was down-regulated, whereas that of 213 (24.04%) mRNAs was up-regulated (Figure 2C). Therefore, down-regulation of translation efficiency was more frequent than up-regulation (genes list in Table S1).

To determine the main functions of the genes whose mRNAs recruitment within polysomes was altered in response to N6L treatment, we performed a gene ontology analysis using the functional annotation clustering analytic modules of EnrichR bioinformatics resources that provides a rank classification of enriched functions based on the determination of *P*-values and enrichment scores [32]. Gene ontology analysis revealed functional pathways corresponding to cell cycle, Fanconi anemia, and RNA transport for the 673 mRNAs translationally repressed (Figure 2E) and functional pathways corresponding to ribosome/translation and oxidative phosphorylation for the 213 mRNAs translationally up-regulated (Figure 2F).

Interestingly, among these 673 translationally down-regulated mRNAs (shown in Figure S2A), 315 were transcriptionally stable (46.8%), encoding notably proteins implicated in phosphatidylinositol biosynthesis (purple panel), and 340 were transcriptionally up-regulated (51%) encoding proteins implicated in mitotic division (green panel) (Figure S2C,D). In addition, among the 213 translationally up-regulated genes (shown in Figure S2B), 73 were transcriptionally stable (34.3%) encoding proteins implicated in translation/ribosomal proteins (blue panel), and 131 were transcriptionally down-regulated (61.5%) encoding proteins implicated in the regulation of cholesterol storage (red panel) (Figure S2E,F). Altogether, these data suggest that the recruitment of these mRNAs into polysomes was controlled upon N6L treatment.

### 3.2. N6L Induces a Global Decrease in mRNA Translation Correlated with a Decrease in EiF3 mRNAs Translation

Because the polysome profiling was indicative of a variation of global translational activity of the cell, we investigated the expression of the mRNAs coding for essential proteins involved in translation regulation that were subjected themselves to a translational regulation as revealed by our functional gene ontology analysis (Figure 2E). Among the 673 mRNA that are down-regulated, several mRNA encode for proteins involved in translation initiation (Figure 2E).

**Figure 3:**
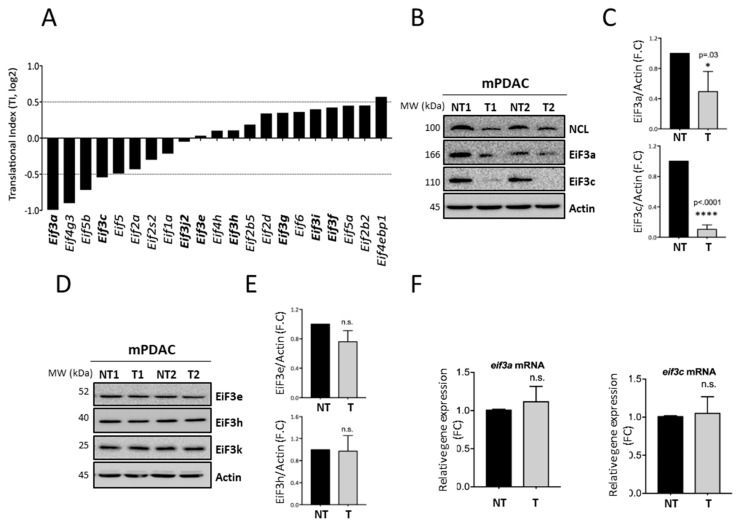
NCL targeting by N6L decreases the translation of mRNAs encoding the canonical subunits of EiF3 factor, Eif3a, and Eif3c. (**A**)—Translation Index (T.I) for eukaryote initiator factors (eIF) mRNA under N6L treatment. (**B**–**E**)—mPDAC cells were treated (T) or not (NT) with N6L for 48 h, following which EiF3 subunits protein expression was analyzed using Western blotting (**B**,**D**) and quantified in relation to actin (**C**,**E**). (**F**)—Validation of relative *eIF3a* and *eIF3c* expression using RT-QPCR in mPDAC treated or not with N6L after normalization to the *actin* housekeeping gene. *p* < 0.05; and n.s. for not statistically significant, as determined by *Student t*-test on final data point.

*EiF3a* mRNA and *EiF3c* mRNA, coding for the essential components of the translation initiation EiF3 complex, were strongly translationally repressed under N6L treatment (TI = −0.99 and −0.54, respectively) while mRNA encoding the other members (*EiF3e-j*) were mostly unchanged (Figure 3A). Using Western blotting analysis, EiF3a and EiF3c protein levels were significantly reduced (Figure 3B,C) while the protein level of the other EiF3 members (EiF3e, h, and k) did not change (Figure 3D,E) upon N6L treatment. Using quantitative PCR, no change was observed in the expression of the *EiF3a and 3c* mRNAs (Figure 3F) in agreement with the transcriptomic analysis (Figure S2). Altogether, these results revealed that N6L decreases the EiF3a and EiF3c expression at the translational level that contributes to the down-regulation of global mRNA translation.

### 3.3. NCL Targeting by N6L Increases 5′TOP mRNA Translation by Activating the mTOR Pathway

**Figure 4:**
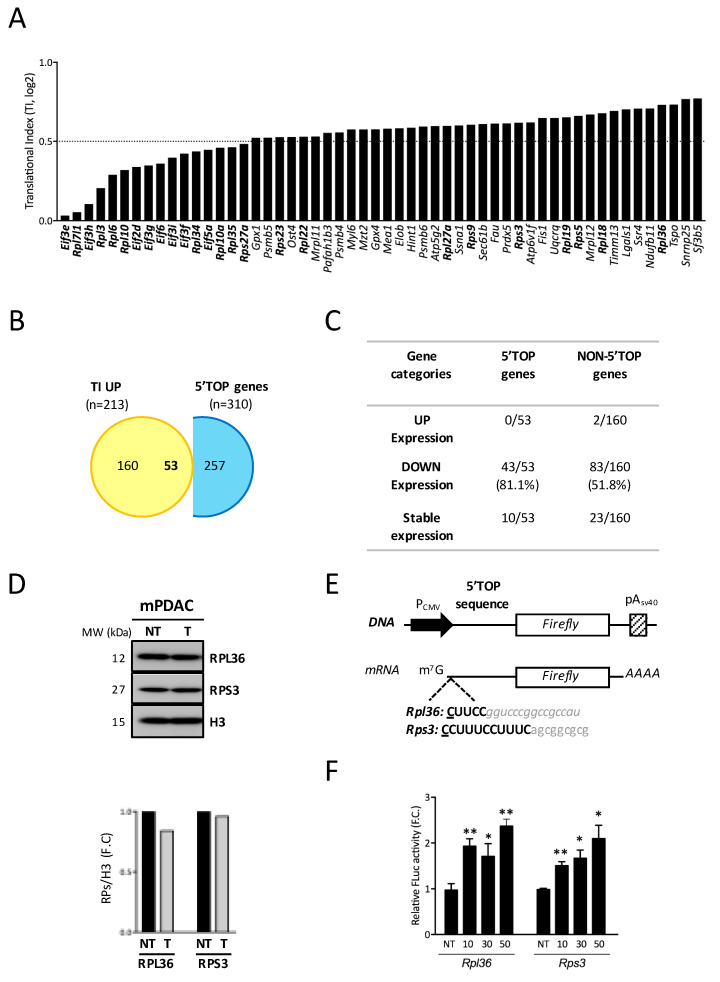
NCL targeting by N6L increases 5′TOP and 5′TISU mRNA translation by activating mTOR pathways. (**A**)—Translation Index (T.I) for 5′TOP-containing mRNA under N6L treatment (**A**, right). (**B**)—Venn diagram comparing 5′TOP-gene overlap between our TI UP mRNAs and 5′TOP gene lists from Gentilella et al. 2017 [33]. (**C**)—Summary table showing the distribution of 5′TOP and non-5′TOP mRNAs according to their transcriptional expression (up, down or stable). (**D**)—mPDAC cells were treated (T) or not (NT) with N6L for 48 h, following which RPL36 and RPS3 expression at the protein level was analyzed using Western blotting (**D**, upper panel) and quantified in relation to H3 (**D**, lower panel). (**E**)—Cartoon showing the design of 5′TOP firefly-luciferase reporter constructs, with the first C of the TOP motif as the +1 transcription start site (TSS). (**F**)—Fold change of firefly-luciferase activities under 5′TOP motifs of *Rpl36* and *Rps3* mRNAs upon 48 h of increasing concentrations of N6L treatment in mPDAC, after normalization to mPDAC NT. * *p* < 0.05; ** *p* < 0.01 and n.s. for not statistically significant, as determined by *Student t*-test on the final data point.

Although the global translation was overall reduced upon N6L treatment, the translatome analysis identified a subset of mRNAs (24.04%) that were enriched in the polysomal fractions (Figure 2C–F). Functional gene ontology analysis of these 213 mRNAs identified genes encoding proteins involved in ribosome biogenesis, translation, or energy metabolism pathways (Figure 2F). In particular, it was very striking to find many mRNAs coding for ribosomal proteins (Figure 4A).

Ribosomal protein transcripts are characterized by the presence of a specific motif in their 5′UTR called 5′ terminal oligopyrimidine (5′TOP) sequence immediately adjacent to the cap structure [34]. This family of 5′TOP mRNAs generally encodes proteins involved in translation initiation, elongation, and termination.

By comparing the mRNAs enriched in polysomes after N6L treatment mRNA to those classified as 5′TOP mRNAs [33], out of these 213 genes enriched in polysome fractions, we identified 53 TOP mRNAs (24.2%) (Figure 4B, Table S1). Not surprisingly, gene ontology analysis of these 53 mRNAs reveals that there are all involved in pathways related to translation, cytoplasmic ribosomal protein, or Cap-dependent translation initiation. In contrast, the set of non-5′TOP is exclusively involved in pathways related to mitochondrial metabolism, including mitochondrial translation, ATP synthesis, or oxidative phosphorylation.

Most of these 53 5′TOP translationally up-regulated mRNAs (81.1%) were transcriptionally down-regulated (Figure 4C). This means that, upon N6L treatment, enrichment in the polysome fraction is not a consequence of a higher accumulation of these mRNAs, but it is rather due to an increase in the recruitment of these mRNAs in the polysomes.

Collectively, these data suggest that NCL targeting by N6L promotes selective recruitment to polysomes of some mRNAs bearing 5′TOP motifs in their 5′UTR. This recruitment maintains translation as seen for RPL36 and RPS3 using Western-blotting analysis (Figure 4D) despite the decreased accumulation of these mRNAs upon N6L treatment.

To determine if the 5′TOP motif plays an active role in this recruitment, we insert the 5′TOP motif of *Rpl36* and *Rps3* mRNAs upstream the *firefly* gene reporters and downstream the CMV promoter (Figure 4E). These plasmids, pFL-5′TOP, were co-transfected with pRL (for normalization) in mPDAC cells treated with increasing doses of N6L. After 24 h, the luciferase activity was quantified to determine if the 5′UTR was required for the increased translation of the chimeric mRNA. Indeed, in the presence of the increasing amount of N6L, the presence of these 5′UTR sequences provided an increasing amount of luciferase activity, suggesting that the presence of this motif increased the recruitment of these mRNAs in the polysomes upon N6L treatment (Figure 4F).

As the 5′TOP motifs are mainly regulated by the mTOR pathway [35], our data suggested that the N6L treatment could activate the mTOR pathway in mPDAC.

**Figure 5:**
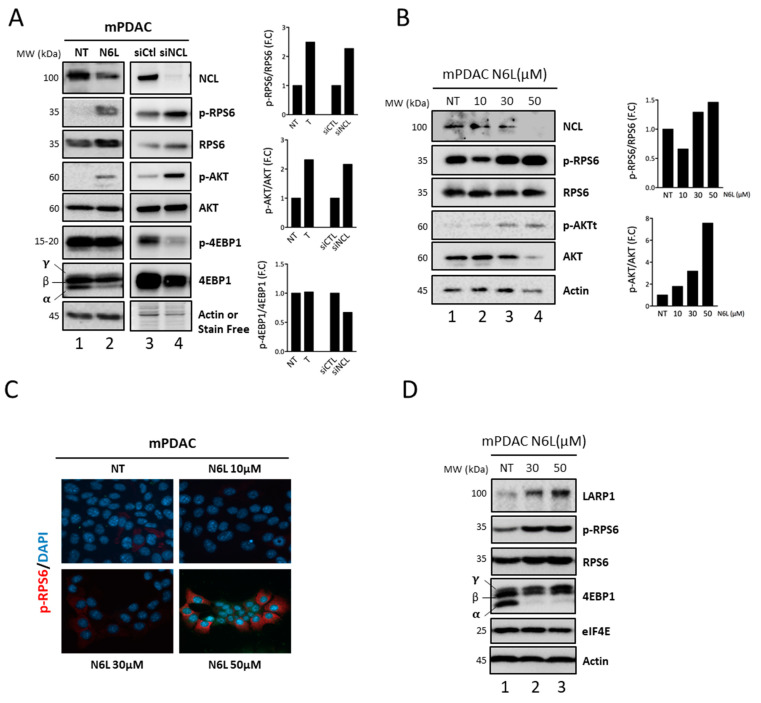
NCL targeting by N6L increases 5′TOP mRNA translation by activating mTOR pathways. (**A**) mTOR pathway activation was analyzed using Western blotting in mPDAC cells, non-treated (NT) or treated with N6L at 50 µM for 48 h (lanes 1,2) or after NCL siRNA depletion (lanes 3,4). **γ** and **β** represent hyper- and **α** hypo-phosphorylated forms of 4EBP1. The phosphorylated-to-total protein ratio was quantified for RPS6, AKT, and 4EBP1. (**B**,**C**)—mTOR pathway activation was analyzed in mPDAC, non-treated (NT), or treated (T) with N6L at increasing doses using Western blotting (**B**) and immunofluorescence staining (**C**) by anti-phosphorylated RPS6 (red) and DAPI for nuclei (blue). (**D**)—Western-blotting analysis of LARP1, RPS6, 4EBP1, and EiF4E expression in mPDAC treated or not with N6L at 30 and 50 µM for 48 h.

Therefore, to determine if the mTOR pathway was activated in mPDAC cells upon N6L treatment, we analyzed the expression/activation of the three primary downstream effectors of mTOR 4E-BP1, RPS6 (a downstream target of S6K), and AKT by performing Western blotting and quantification of the phosphorylated-to-total form ratios of RPS6, 4EBP1 and AKT accumulations (Figure 5A–D).

We showed that the N6L treatment induces strong phosphorylation of RPS6, 4EBP1 (**γ** and **β** forms), and AKT, compared to non-treated mPDAC cells (Figure 5A; lanes 1,2) while the expression of NCL protein is decreased. We then determined whether NCL expression modulates the mTOR pathway. Indeed, the activation of mTOR, via the hyper-phosphorylation of RPS6, AKT, and 4EBP1, was also observed when NCL was down-regulated by siRNA (Figure 5A, lanes 3,4). In addition, the phosphorylation of RPS6 and AKT occurred in an N6L dose-dependent manner as revealed by Western blotting (Figure 5B) and immunofluorescence analysis of phosphorylated RPS6 (Figure 5C).

Collectively, these results showed that NCL inhibition, either through siRNA or with N6L treatment, causes activation of the mTOR pathway in mPDAC cells.

The translation of 5′TOP mRNAs is regulated by the binding of La-related protein 1 (LARP1) to the cap and TOP sequence [33,36,37]. Indeed, Western-blotting results showed an accumulation of LARP1 in an N6L dose-dependent manner (Figure 5D).

In sum, these data demonstrate that the N6L treatment modified the translatome of treated PDAC cells through the activation of the mTOR pathway, thereby allowing the translation of a specific set of mRNAs involved in the translational and metabolic pathways.

### 3.4. Combinations of N6L and mTORi Are Synergistic on PDAC Cell Growth and Viability Inhibition

The activation of the mTOR pathway by N6L induces an increase in translation of 5′TOP mRNAs, which may contribute to a decrease in efficiency of the N6L treatment on cell viability and proliferation. We tested the effects of the combination of the mTOR inhibitors with N6L in human and murine PDAC models.

We used the allosteric mTORC1 inhibitor rapamycin (RAPA, Sirolimus) and the ATP-competitive mTOR kinase inhibitors AZD2014 (Vistusertib) and INK128 (Sapanisertib), which inhibit both mTORC1 and mTORC2 [38,39].

**Figure 6:**
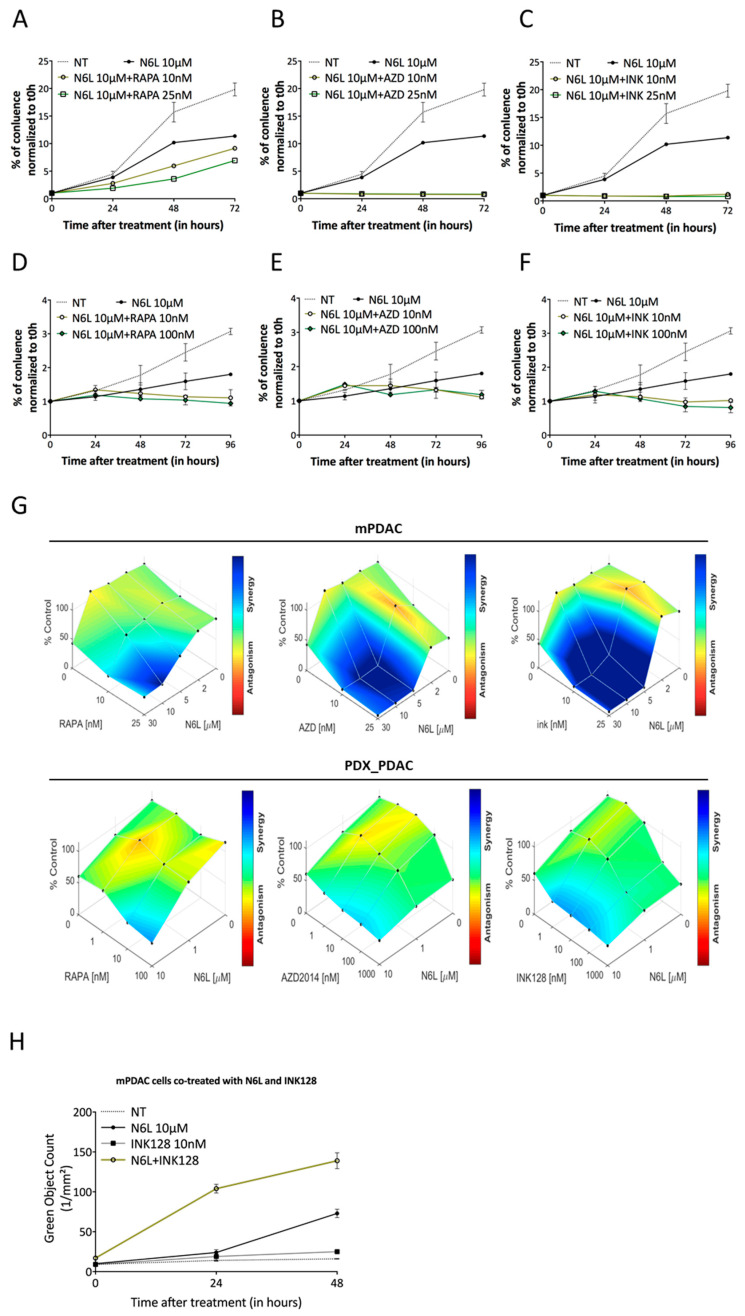

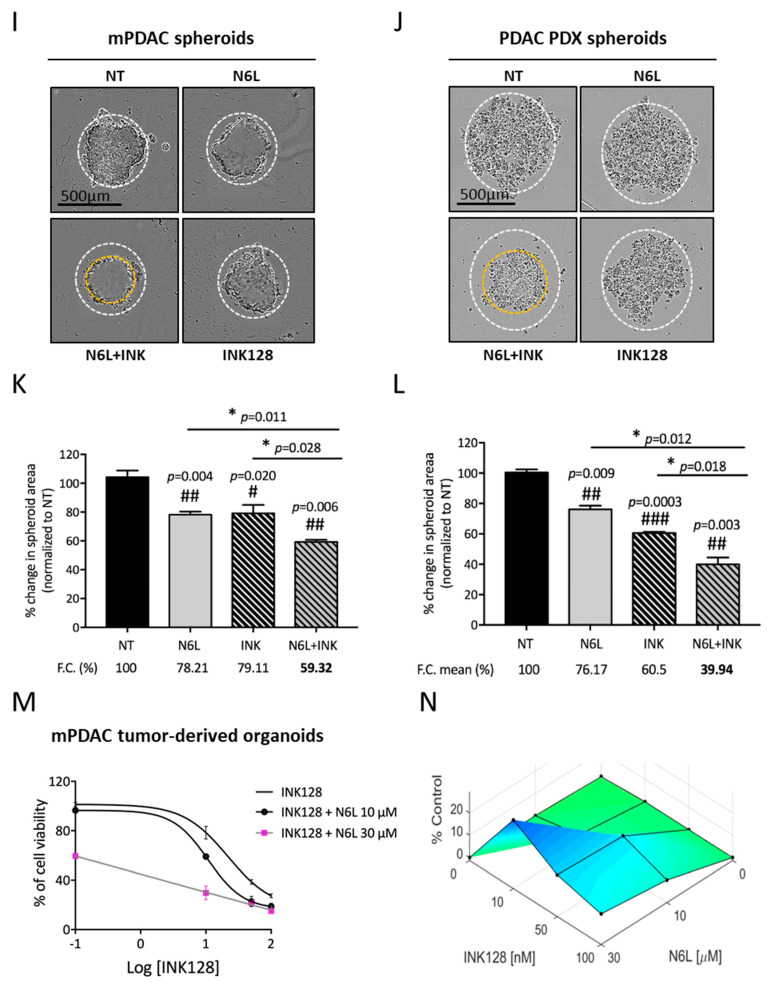
N6L and mTORi combination acts synergistically to decrease PDAC cell and spheroid growth and organoid viability. (**A**–**C**), mPDAC cell lines were seeded in 96-well plates and allowed to adhere overnight. Cells were treated with 10 or 25 nM of rapamycin (RAPA, **A**), AZD2014 (AZD, **B**), or INK128 (INK, **C**) in combinations with N6L at 10 µM over 72 h. (**D**–**F**), PDAC PDX (PAN014) cell lines were seeded in 96-well plates and allowed to adhere for 2 days. Cells were treated with 1, 10, or 100nM of RAPA, AZD, or INK in combinations with N6L at 10 µM over 96 h. Cell growth was assessed by IncuCyte ZOOM^®^ (Michigan, USA) live-imaging. The AUC (area under curves) was calculated by IncuCyte ZOOM^®^ live-imaging software. Growth for treated cells was normalized to untreated cells on the same plate. (**G**)—Synergy/antagonism calculation. The contour views from the LOEWE model were selected as graphical outputs for the synergy distribution. (**H**), Apoptosis induction assay is performed with mPDAC incubated with 10 µM of N6L alone or in combination with 10 nM of INK128 using InCucyte ZOOM live imaging system. Data are represented showing the number of caspase-3/7 positive cells (green color object) per surface (mm^2^). (**I**,**J**), Representative images of mPDAC (**I**) and PDAC PDX (**J**) spheroids non-treated (NT) or treated with N6L alone or combined with INK128 over 96 h. (**K**–**L**), Spheroid area was calculated using ImageJ software, and data were normalized to control for combinations of N6L and INK128. (**M**), mPDAC organoid viability assessed with MTS assay upon treatment with INK128 alone or in combination with N6L at 10 µM and 30 µM. (**N**), Contour view showing the synergy distribution from MTS assay on mPDAC organoid. Scale bar, 500 µm. (#) if comparing each treatment to NT, (*) if comparing the combination to a single treatment. * *p* < 0.05; ** *p* < 0.01; and n.s. for not statistically significant, as determined by Student’s *t*-test on the final data point.

Two concentrations of RAPA, AZD2014, or INK128 were tested alone or in combination with N6L for mPDAC confluence/growth assay over 72 h using IncuCyte ZOOM^®^ live-imaging (Figure 6 and Figure S3). As previously described in Figure S1, N6L induced a decrease in cell growth in a dose-dependent manner, whereas mTORi alone had no or modest effects on mPDAC cell growth at the tested concentrations (10 and 25 nM) (Figure S3A–C). However, the combination of both mTOR inhibitors and N6L had strong inhibitory effects on mPDAC cell growth (Figure 6A–C), suggesting an additive or synergistic effect between the two molecules. Using the Combenefit^®^ software [23] based on the LOEWE method, we demonstrated that the combination of N6L (5 or 10 µM) and mTORi had a strong synergistic effect on the mPDAC growth inhibition (Figure 6G; upper panels).

We then further validated these combinations in patient-derived xenograft (PDX) models and other preclinical cell lines. Whereas mTORi alone has no or modest effects on PDX cell growth at the tested concentrations (Figure S3D–F), except for INK128 at a high concentration of 100 nM), the combination of RAPA, AZD2014, or INK128 with N6L was superior to single-agent treatment (Figure 6D–F), and this effect on PDX growth inhibition was synergistic (Figure 6G, lower panels).

The same results were obtained in human PDAC cell lines MiaPaca-2 (Figure S4A–C) and Panc-1 (Figure S5A–C) upon N6L/mTORi combination.

As N6L treatment impairs the cell viability of mPDAC cells (Figure S6A), we wanted to determine if the death of the cells was due to caspase-dependent apoptosis as previously described [9,17]. Apoptosis induction assay was performed using the IncuCyte ZOOM^®^ live-imaging. A fluorescent reagent, activated when effector caspases 3/7 were activated, allowed the device to count the number of apoptotic cells per mm^2^ every 2 h during 72 h. We observed a strong and significant increase in caspase-dependent apoptosis in mPDAC-treated cells in a dose-dependent manner compared to non-treated cells (Figure S6B). In addition, the morphology of the N6L-treated cells appears different from that of non-treated cells.

Upon N6L and mTORi dual treatment, we also observed a strong increase in caspase-dependent apoptosis in mPDAC-treated cells with N6L combined to the dual mTORi INK128 (Figure 6H).

To accurately predict the efficacy of these new drug combinations for PDAC therapy, we tested them on three-dimensional culture models by establishing spheroids and PDAC tumor-derived organoid cultures. To this end, we allowed PDAC and PDX cells to form spheroid, as previously described [40]. Then, we treated them with different drugs concentrations of N6L or INK128, alone or in combinations over 96 h, using IncuCyte ZOOM^®^ live-imaging (Figure 6I–L). To evaluate the impact of the drug combination on spheroid growth, their area was calculated using ImageJ software. The results showed that the spheroid area decreased by 22% and 21%, upon N6L or INK128 alone, respectively (Figure 6I–K), while when INK128 is combined with N6L treatment, the mPDAC spheroid area decreased more than 41% (*p* = 0.006). The same results were obtained for PDX spheroids (Figure 6J), MiaPaca-2 (Figure S4D,E), and Panc-1 (Figure S5D,E) cell lines. In PDX models, the combination of N6L and INK128 significantly decreased the PDX spheroid area by more than 60% (*p =* 0.003) comparing to non-treated conditions (Figure 6L).

We also tested the combination of N6L and INK128 on mPDAC tumor-derived organoid viability (Figure 6M,N). The organoid cultures were established from mPDAC tumors generated in mice as previously described [22,41]. An analysis of MTS data by the LOEWE method in the PDAC-derived organoids models demonstrated that the combination of N6L and INK128 had an inhibitory synergistic effect on the cell viability (Figure 6M,N).

**Figure 7:**
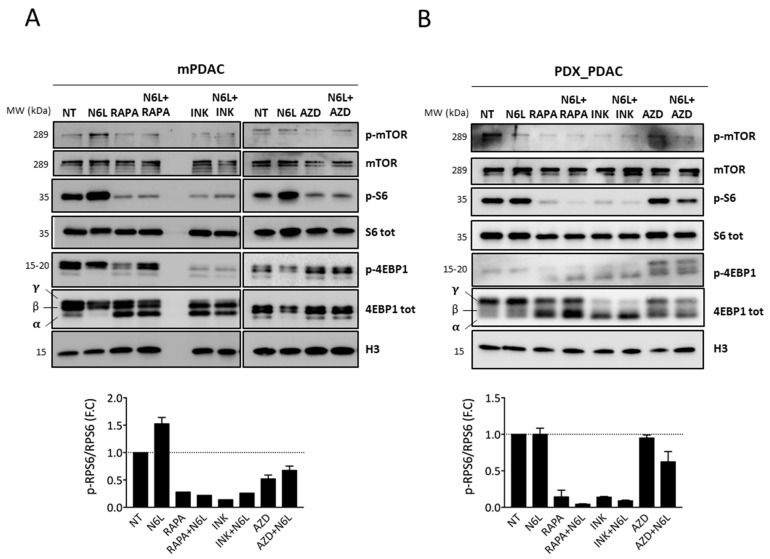
N6L combined to mTORi treatment inactivate mTOR pathway by decreasing RPS6 and 4EBP1 phosphorylation. mTOR pathway inhibition was analyzed using Western blotting in mPDAC (**A**) and PDX_PDAC (**B**) cells non-treated (NT) or treated with N6L, RAPA, INK128, and AZD2014 alone or in combination (N6L+RAPA, N6L+INK, or N6L+AZD).

Mechanistically, we confirmed by Western-blotting analysis the inactivation of mTOR pathway upon the N6L and mTORi dual treatment (Figure 7A,B), where a strong decrease in RPS6 and 4EBP1 phosphorylation in mPDAC and PDX cells is observed. We obtained the same results on the other human PDAC cell lines MiaPaca and Panc (Figure S5F and Figure S6F, respectively). Of note, the dual mTORC1/mTORC2 INK128 inhibitor seemed to have a strong effect in comparison to the other mTOR inhibitors when combined with N6L.

Collectively, these results demonstrate that NCL targeting by N6L treatment and inhibition of the TOR pathway act synergistically to inhibit PDAC cell growth.

## 4. Discussion

Nucleolin (NCL) overexpression is associated with a poor prognostic for many cancers, including pancreatic cancer. PDAC remains one of the most aggressive and lethal diseases because of the limited therapeutic options. The nucleolin aptamer N6L shows antitumor effects on breast cancer [17], glioblastoma [19], non-small cell lung carcinoma [20], and PDAC [9,21].

NCL is a multifunctional protein that is required for cell growth and proliferation. The inhibition of NCL with aptamers such as N6L, AS1411, or through silencing with siRNA induces a profound remodeling of the cell (and nucleus) morphology with a strong effect on the cell cycle progression and on genes expressions [10,14]. Our study shows that PDAC cells treated with NCL inhibitors activate pathways that allow the production of proteins involved in translation, despite the global translational repression indicating that the cells are trying to overcome the inhibition of NCL function. The activation of the mTOR signaling pathway is part of this cellular response to NCL inhibition to help the cell to survive despite the inhibition of many of the vital functions of NCL. The synergistic effect of NCL and mTOR inhibitors shows that the activation of the mTOR pathway is important for the survival of the cells when NCL is inhibited.

We found that although N6L strongly inhibits global translation, some mRNAs escape this global translational shutdown (Figure 2). The expression of these mRNAs that continue to be recruited in polysomes in N6L-treated cells was either stable or lower (Figure S2), indicating that this increased recruitment in polysomes was not the consequence of an increase in their transcription but rather the consequence of regulation at the translational level. These translationally up-regulated mRNAs are mostly implicated in cell metabolism and translation machinery.

Many of these mRNAs recruited in the polysomes under N6L treatment contains a 5′-Terminal oligopyrimidine (5′TOP) in their 5′UTR and encodes for ribosomal proteins and for factors regulating translation.

The 5′TOP motif begins with a C nucleotide directly adjacent to the cap structure, followed by a series of approximately 4–15 pyrimidines, often followed by a G-rich region [34]. The 5′TOP motif is highly conserved and is found in all human ribosomal proteins as well as non-ribosomal proteins involved in translation [42,43]. This shared TOP motif allows cells to quickly modulate the expression of proteins involved in ribosome production and protein synthesis in response to changes in cellular homeostasis. We hypothesize that the cells activate the translation of these 5′TOP mRNAs to counteract the negative effect of N6L on cell survival. Mechanistically, the translation of these 5′UTR motifs is regulated by the mTOR pathway.

mTOR pathway is crucial to control cell growth and survival in physiological as well as pathological conditions. It acts in two distinct mTOR complexes, mTORC1 and mTORC2, which differ in associated proteins and by sensitivity to rapamycin and its derivatives [44]. The major function of mTORC1 is the promotion of cellular growth and proliferation via increasing protein synthesis and inhibition of autophagy. Functions of mTORC2 are less well studied and include organization of the actin cytoskeleton, control of cellular metabolism, and anti-apoptotic properties via stimulation of the AKT-FOXO pathway.

The inhibition of the mTORC1 pathway leads to a strong decrease in the translation of transcripts containing the 5′TOP motif [35,45]. However, how mTOR regulates 5′TOP mRNA translation remains not completely understood.

Many factors have been proposed as trans-acting factors that could regulate (positively or negatively) the translation of 5′TOP mRNA, including the T-cell intracellular antigen-1 (TIA1) and TIA-1-related (TIAR) proteins, miR-10A, 4EBP1, and LARP1 [46]. mTORC1 enhance 5′TOP mRNA translation by two mechanisms: phosphorylation of 4EBP1 and/or LARP1 [35,37,39].

Under mTOR activation, 4EBP1 is phosphorylated, which prevents 4EBP1 from disrupting the interaction of eIF4E and the mRNA cap, and has been shown to selectively enhance 5′TOP mRNA translation [35,39]. The role of LARP1 in the regulation of TOP mRNAs remains controversial. While LARP1 was shown to regulate TOP mRNA stability, it has also been described as a positive or negative regulator of TOP mRNA translation, depending on the context and the energy status of the cells [47]. LARP1 interacts with the raptor and is phosphorylated by mTORC1, which is thought to modulate its mRNA-binding activity [45,48]. In our study, the phosphorylation of 4EBP1, RPS6, and AKT and the accumulation of LARP1 upon N6L treatment enhance the 5′TOP mRNA translation show that the mTOR pathway is activated and that LARP1 could act as a positive regulator.

The mTOR pathway is reported to be aberrantly active in several cancers, including PDAC [49,50], in part due to mutations in upstream regulatory molecules including PTEN, AKT, and TSC1/2. Most PDAC cancers have RAS mutations leading to activation of the MEK/ERK pathway, which can inactivate TSC1/2 and thereby activate TORC1. Therefore, mTOR is a relevant target for the treatment of PDAC [49]. Several types of mTOR inhibitors such as rapamycin, its rapalogs, and dual mTORC1/mTORC2 inhibitors have been examined in various cancer models, including PDAC [49,51]. However, the effects of mTOR inhibitors used as monotherapy in clinical trials have not shown significant effects and are sometimes dampened by several resistance mechanisms [52]. Combined therapies with mTOR inhibitors and other pathway inhibitors or conventional therapies are under investigation in preclinical and clinical trials in different tumor types [53]. Hence, novel therapeutic strategies based on mTOR inhibition still need to be developed. In PDAC, there is an increased need for new therapeutic strategies with better efficacy and less toxicity.

In our study, NCL targeting by N6L enhances the mTOR pathway-inducing reprogramming of the translatome by an increase in specific mRNAs (including 5′TOP mRNAs) in the polysomes while the translation of most of the mRNAs is strongly down-regulated. This specific recruitment of mRNAs coding for ribosomal proteins and for factors required for the regulation of translation could be involved in mechanisms put in place by the cell in reaction to N6L to try to better resist the inhibitory effects of the drugs.

Indeed, when we combined N6L with mTOR inhibitors, we observed a synergistic inhibitory effect in different 2D and 3D preclinical models including, PDX and organoid-derived tumors that faithfully reproduce the molecular features of PDAC human tumors, display a high predictive value for clinical efficacy in patients and are critically needed for the development of new treatments. We used two classes of mTORi, allosteric mTORC1 inhibitor rapamycin (RAPA, Sirolimus) and dual mTORC1/mTORC2 inhibitors AZD2014 (Vistusertib) and INK128 (Sapanisertib) [38,39]. Furthermore, we showed that this synergistic effect of inhibiting cell proliferation and viability is maintained even when the PDAC cells are co-treated with low doses of N6L and mTORi (data not shown) that can help to prevent side effects due to mTORi toxicity in the clinic by reducing mTORi doses. In addition, we confirmed by Western-blotting analysis the inactivation of the mTOR pathway upon the N6L and mTORi dual treatment.

## 5. Conclusions

In this study, we show that the NCL aptamer’s strong inhibition of PDAC cell growth is coupled with the reprogramming of the translational machinery through the activation of the mTOR signaling pathway. We further demonstrate that NCL targeting by N6L treatment sensitizes PDAC cells to mTOR inhibitors. We propose a novel therapeutic strategy based on mTOR inhibition combined with NCL targeting by N6L to treat PDAC.

## Data Availability

The RNASeq gene expression data and raw fastq files are available on the GEO repository (www.ncbi.nlm.nih.gov/geo/, accessed on 15 September 2021) under accession number: GSE184857, and the data presented in this study are available on request from the corresponding author.

## Supplementary Materials

The following are available online at https://www.mdpi.com/article/10.3390/cancers13194957/s1, Table S1: RNAseq results of control and N6L-treated PDAC cells. Translational index calculation and analysis of 5′TOP-containing mRNAS. Table S2: list of antibodies used in the studies. Figure S1: NCL targeting by N6L decreases mPDAC cell proliferation by inducing apoptosis. Figure S2: NCL targeting by N6L dysregulates mPDAC cell translation. Figure S3: The impact of mTOR inhibitors treatment on mPDAC and PDX cell growth. Figure S4: Synergistic effects of N6L/mTORi combination on MiaPaca cells. Figure S5: Synergistic effects of N6L/mTORi combination on Panc cells Figure S6: Originals western blot images.

## References

[1] Kamisawa T., Wood L.D., Itoi T., Takaori K. (2016). Pancreatic Cancer. Lancet.

[2] Søreide K., Primavesi F., Labori K.J., Watson M.M., Stättner S. (2019). Molecular Biology in Pancreatic Ductal Adenocarcinoma: Implications for Future Diagnostics and Therapy. Eur. Surg..

[3] Bachet J.-B., Hammel P., Desramé J., Meurisse A., Chibaudel B., André T., Debourdeau P., Dauba J., Lecomte T., Seitz J.-F. (2017). Nab-Paclitaxel plus Either Gemcitabine or Simplified Leucovorin and Fluorouracil as First-Line Therapy for Metastatic Pancreatic Adenocarcinoma (AFUGEM GERCOR): A Non-Comparative, Multicentre, Open-Label, Randomised Phase 2 Trial. Lancet Gastroenterol. Hepatol..

[4] Gunturu K.S., Yao X., Cong X., Thumar J.R., Hochster H.S., Stein S.M., Lacy J. (2013). FOLFIRINOX for Locally Advanced and Metastatic Pancreatic Cancer: Single Institution Retrospective Review of Efficacy and Toxicity. Med. Oncol..

[5] Guo X., Xiong L., Yu L., Li R., Wang Z., Ren B., Dong J., Li B., Wang D. (2014). Increased Level of Nucleolin Confers to Aggressive Tumor Progression and Poor Prognosis in Patients with Hepatocellular Carcinoma after Hepatectomy. Diagn. Pathol..

[6] Marcel V., Catez F., Berger C.M., Perrial E., Plesa A., Thomas X., Mattei E., Hayette S., Saintigny P., Bouvet P. (2017). Expression Profiling of Ribosome Biogenesis Factors Reveals Nucleolin as a Novel Potential Marker to Predict Outcome in AML Patients. PLoS ONE.

[7] Zhao H., Huang Y., Xue C., Chen Y., Hou X., Guo Y., Zhao L., Hu Z.H., Huang Y., Luo Y. (2013). Prognostic Significance of the Combined Score of Endothelial Expression of Nucleolin and CD31 in Surgically Resected Non-Small Cell Lung Cancer. PLoS ONE.

[8] Qiu W., Zhou F., Zhang Q., Sun X., Shi X., Liang Y., Wang X., Yue L. (2013). Overexpression of Nucleolin and Different Expression Sites Both Related to the Prognosis of Gastric Cancer. APMIS.

[9] Gilles M.-E., Maione F., Cossutta M., Carpentier G., Caruana L., Di Maria S., Houppe C., Destouches D., Shchors K., Prochasson C. (2016). Nucleolin Targeting Impairs the Progression of Pancreatic Cancer and Promotes the Normalization of Tumor Vasculature. Cancer Res..

[10] Ugrinova I., Petrova M., Chalabi-Dchar M., Bouvet P. (2018). Multifaceted Nucleolin Protein and Its Molecular Partners in Oncogenesis. Advances in Protein Chemistry and Structural Biology.

[11] Hovanessian A.G., Puvion-Dutilleul F., Nisole S., Svab J., Perret E., Deng J.-S., Krust B. (2000). The Cell-Surface-Expressed Nucleolin Is Associated with the Actin Cytoskeleton. Exp. Cell Res..

[12] Koutsioumpa M., Papadimitriou E. (2014). Cell Surface Nucleolin as a Target for Anti-Cancer Therapies. Recent Pat. Anticancer Drug Discov..

[13] Farin K., Schokoroy S., Haklai R., Cohen-Or I., Elad-Sfadia G., Reyes-Reyes M.E., Bates P.J., Cox A.D., Kloog Y., Pinkas-Kramarski R. (2011). Oncogenic Synergism between ErbB1, Nucleolin, and Mutant Ras. Cancer Res..

[14] Kumar S., Gomez E.C., Chalabi-Dchar M., Rong C., Das S., Ugrinova I., Gaume X., Monier K., Mongelard F., Bouvet P. (2017). Integrated Analysis of MRNA and MiRNA Expression in HeLa Cells Expressing Low Levels of Nucleolin. Sci. Rep..

[15] Krust B., El Khoury D., Nondier I., Soundaramourty C., Hovanessian A.G. (2011). Targeting Surface Nucleolin with Multivalent HB-19 and Related Nucant Pseudopeptides Results in Distinct Inhibitory Mechanisms Depending on the Malignant Tumor Cell Type. BMC Cancer.

[16] Destouches D., El Khoury D., Hamma-Kourbali Y., Krust B., Albanese P., Katsoris P., Guichard G., Briand J.P., Courty J., Hovanessian A.G. (2008). Suppression of Tumor Growth and Angiogenesis by a Specific Antagonist of the Cell-Surface Expressed Nucleolin. PLoS ONE.

[17] Destouches D., Page N., Hamma-Kourbali Y., Machi V., Chaloin O., Frechault S., Birmpas C., Katsoris P., Beyrath J., Albanese P. (2011). A Simple Approach to Cancer Therapy Afforded by Multivalent Pseudopeptides That Target Cell-Surface Nucleoproteins. Cancer Res..

[18] Birmpas C., Briand J.P., Courty J., Katsoris P. (2012). Nucleolin Mediates the Antiangiogenesis Effect of the Pseudopeptide N6L. BMC Cell Biol..

[19] Benedetti E., Antonosante A., d’Angelo M., Cristiano L., Galzio R., Destouches D., Florio T.M., Dhez A.C., Astarita C., Cinque B. (2015). Nucleolin Antagonist Triggers Autophagic Cell Death in Human Glioblastoma Primary Cells and Decreased in Vivo Tumor Growth in Orthotopic Brain Tumor Model. Oncotarget.

[20] Ramos K.S., Moore S., Runge I., Tavera-Garcia M.A., Cascone I., Courty J., Reyes-Reyes E.M. (2020). The Nucleolin Antagonist N6L Inhibits LINE1 Retrotransposon Activity in Non-Small Cell Lung Carcinoma Cells. J. Cancer.

[21] Sanhaji M., Göring J., Couleaud P., Aires A., Cortajarena A.L., Courty J., Prina-Mello A., Stapf M., Ludwig R., Volkov Y. (2019). The Phenotype of Target Pancreatic Cancer Cells Influences Cell Death by Magnetic Hyperthermia with Nanoparticles Carrying Gemicitabine and the Pseudo-Peptide NucAnt. Nanomed. Nanotechnol. Biol. Med..

[22] Boj S.F., Hwang C.-I., Baker L.A., Chio I.I.C., Engle D.D., Corbo V., Jager M., Ponz-Sarvise M., Tiriac H., Spector M.S. (2015). Organoid Models of Human and Mouse Ductal Pancreatic Cancer. Cell.

[23] Di Veroli G.Y., Fornari C., Wang D., Mollard S., Bramhall J.L., Richards F.M., Jodrell D.I. (2016). Combenefit: An Interactive Platform for the Analysis and Visualization of Drug Combinations. Bioinformatics.

[24] David A., Dolan B.P., Hickman H.D., Knowlton J.J., Clavarino G., Pierre P., Bennink J.R., Yewdell J.W. (2012). Nuclear Translation Visualized by Ribosome-Bound Nascent Chain Puromycylation. J. Cell Biol..

[25] Bash-Imam Z., Thérizols G., Vincent A., Lafôrets F., Espinoza M.P., Pion N., Macari F., Pannequin J., David A., Saurin J.-C. (2017). Translational Reprogramming of Colorectal Cancer Cells Induced by 5-Fluorouracil through a MiRNA-Dependent Mechanism. Oncotarget.

[26] Jourdren L., Bernard M., Dillies M.-A., Le Crom S. (2012). Eoulsan: A Cloud Computing-Based Framework Facilitating High Throughput Sequencing Analyses. Bioinformatics.

[27] Dobin A., Davis C.A., Schlesinger F., Drenkow J., Zaleski C., Jha S., Batut P., Chaisson M., Gingeras T.R. (2013). STAR: Ultrafast Universal RNA-Seq Aligner. Bioinformatics.

[28] Li H., Handsaker B., Wysoker A., Fennell T., Ruan J., Homer N., Marth G., Abecasis G., Durbin R. (2009). 1000 Genome Project Data Processing Subgroup The Sequence Alignment/Map Format and SAMtools. Bioinformatics.

[29] Anders S., Pyl P.T., Huber W. (2015). HTSeq--a Python Framework to Work with High-Throughput Sequencing Data. Bioinformatics.

[30] Love M.I., Huber W., Anders S. (2014). Moderated Estimation of Fold Change and Dispersion for RNA-Seq Data with DESeq2. Genome Biol..

[31] Sonenberg N., Hinnebusch A.G. (2009). Regulation of Translation Initiation in Eukaryotes: Mechanisms and Biological Targets. Cell.

[32] Kuleshov M.V., Diaz J.E.L., Flamholz Z.N., Keenan A.B., Lachmann A., Wojciechowicz M.L., Cagan R.L., Ma’ayan A. (2019). ModEnrichr: A Suite of Gene Set Enrichment Analysis Tools for Model Organisms. Nucleic Acids Res..

[33] Gentilella A., Morón-Duran F.D., Fuentes P., Zweig-Rocha G., Riaño-Canalias F., Pelletier J., Ruiz M., Turón G., Castaño J., Tauler A. (2017). Autogenous Control of 5′TOP MRNA Stability by 40S Ribosomes. Mol. Cell.

[34] Meyuhas O., Kahan T. (2015). The Race to Decipher the Top Secrets of TOP MRNAs. Biochim. Biophys. Acta BBA Gene Regul. Mech..

[35] Thoreen C.C., Chantranupong L., Keys H.R., Wang T., Gray N.S., Sabatini D.M. (2012). A Unifying Model for MTORC1-Mediated Regulation of MRNA Translation. Nature.

[36] Aoki K., Adachi S., Homoto M., Kusano H., Koike K., Natsume T. (2013). LARP1 Specifically Recognizes the 3′ Terminus of Poly(A) MRNA. FEBS Lett..

[37] Fonseca B.D., Lahr R.M., Damgaard C.K., Alain T., Berman A.J. (2018). LARP1 on TOP of Ribosome Production. Wiley Interdiscip. Rev. RNA.

[38] Pike K.G., Malagu K., Hummersone M.G., Menear K.A., Duggan H.M.E., Gomez S., Martin N.M.B., Ruston L., Pass S.L., Pass M. (2013). Optimization of Potent and Selective Dual MTORC1 and MTORC2 Inhibitors: The Discovery of AZD8055 and AZD2014. Bioorg. Med. Chem. Lett..

[39] Hsieh A.C., Liu Y., Edlind M.P., Ingolia N.T., Janes M.R., Sher A., Shi E.Y., Stumpf C.R., Christensen C., Bonham M.J. (2012). The Translational Landscape of MTOR Signalling Steers Cancer Initiation and Metastasis. Nature.

[40] Ware M.J., Colbert K., Keshishian V., Ho J., Corr S.J., Curley S.A., Godin B. (2016). Generation of Homogenous Three-Dimensional Pancreatic Cancer Cell Spheroids Using an Improved Hanging Drop Technique. Tissue Eng. Part C Methods.

[41] Tiriac H., Belleau P., Engle D.D., Plenker D., Deschênes A., Somerville T.D.D., Froeling F.E.M., Burkhart R.A., Denroche R.E., Jang G.-H. (2018). Organoid Profiling Identifies Common Responders to Chemotherapy in Pancreatic Cancer. Cancer Discov..

[42] Yamashita R., Suzuki Y., Takeuchi N., Wakaguri H., Ueda T., Sugano S., Nakai K. (2008). Comprehensive Detection of Human Terminal Oligo-Pyrimidine (TOP) Genes and Analysis of Their Characteristics. Nucleic Acids Res..

[43] Levy S., Avni D., Hariharan N., Perry R.P., Meyuhas O. (1991). Oligopyrimidine Tract at the 5′ End of Mammalian Ribosomal Protein MRNAs Is Required for Their Translational Control. Proc. Natl. Acad. Sci. USA.

[44] Sabatini D.M. (2006). MTOR and Cancer: Insights into a Complex Relationship. Nat. Rev. Cancer.

[45] Philippe L., van den Elzen A.M.G., Watson M.J., Thoreen C.C. (2020). Global Analysis of LARP1 Translation Targets Reveals Tunable and Dynamic Features of 5′TOP Motifs. Proc. Natl. Acad. Sci. USA.

[46] Patursky-Polischuk I., Kasir J., Miloslavski R., Hayouka Z., Hausner-Hanochi M., Stolovich-Rain M., Tsukerman P., Biton M., Mudhasani R., Jones S.N. (2014). Reassessment of the Role of TSC, MTORC1 and MicroRNAs in Amino Acids-Meditated Translational Control of TOP MRNAs. PLoS ONE.

[47] Al-Ashtal H.A., Rubottom C.M., Leeper T.C., Berman A.J. (2021). The LARP1 La-Module Recognizes Both Ends of TOP MRNAs. RNA Biol..

[48] Hong S., Freeberg M.A., Han T., Kamath A., Yao Y., Fukuda T., Suzuki T., Kim J.K., Inoki K. (2017). LARP1 Functions as a Molecular Switch for MTORC1-Mediated Translation of an Essential Class of MRNAs. eLife.

[49] Tian T., Li X., Zhang J. (2019). MTOR Signaling in Cancer and MTOR Inhibitors in Solid Tumor Targeting Therapy. Int. J. Mol. Sci..

[50] Bellizzi A.M., Bloomston M., Zhou X.-P., Iwenofu O.H., Frankel W.L. (2010). The MTOR Pathway Is Frequently Activated in Pancreatic Ductal Adenocarcinoma and Chronic Pancreatitis. Appl. Immunohistochem. Mol. Morphol..

[51] Hassan Z., Schneeweis C., Wirth M., Veltkamp C., Dantes Z., Feuerecker B., Ceyhan G.O., Knauer S.K., Weichert W., Schmid R.M. (2018). MTOR Inhibitor-Based Combination Therapies for Pancreatic Cancer. Br. J. Cancer.

[52] Guri Y., Hall M.N. (2016). MTOR Signaling Confers Resistance to Targeted Cancer Drugs. Trends Cancer.

[53] Clinical Trials. https://clinicaltrials.gov/ct2/results?cond=mTOR+inhibitors&term=&cntry=&state=&city=&dist=.

