# Supplementary material for: Nucleolin Aptamer N6L Reprograms the Translational Machinery and Acts Synergistically with mTORi to Inhibit Pancreatic Cancer Proliferation
Source: Cancers (Basel). 2021 Oct 1;13(19):4957. doi: 10.3390/cancers13194957 (PMC8508287; doi:10.3390/cancers13194957)
Supplement: Supplementary file 1 [file cancers-13-04957-s001.zip › cancers-1318085-SI/Chalabi et al_Supplementary data_final_30092021.pdf]

Supplementary informations

Nucleolin Aptamer N6L Reprograms the Translational Machinery and Acts Synergistically with mTORi to Inhibit Pancreatic Cancer Proliferation

Mounira Chalabi-Dchar [1], Elisabeth Cruz [1], Hichem C. Mertani [1], Jean-Jacques Diaz [1], José Courty [2], Ilaria Cascone [2] and Philippe Bouvet [1,3,*]

1. Centre de Recherche en Cancérologie de Lyon, Université de Lyon 1, Inserm U1052, CNRS UMR5286 Centre Léon Bérard, F-69373 Lyon Cedex 08, France; Mounira.CHALABI@lyon.unicancer.fr (M.C.-D.); Elizabeth.CRUZGOMEZ@lyon.unicancer.fr (E.C.); hichem.mertani@lyon.unicancer.fr (H.C.M.); JeanJacques.DIAZ@lyon.unicancer.fr (J.-J.D.)
2. INSERM, Institut Mondor de Recherche Biomédicale (IMRB), Université Paris-Est Créteil, F-94010 Créteil, France; courty@u-pec.fr J.C.); ilaria.cascone@u-pec.fr (I.C.)
3. Ecole Normale Supérieure de Lyon, Université de Lyon 1, F-69007 Lyon, France

* Correspondence: pbouvet@ens-lyon.fr

Corresponding author, Philippe Bouvet, Centre de Recherche en Cancérologie de Lyon, Université de Lyon, Centre Léon Bérard, F-69373 Lyon Cedex 08, France, pbouvet@ens-lyon.fr

Running title: Synergistic action of nucleolin aptamers and mTORi

Keywords: Nucleolin, pancreatic cancer, translation, mTOR inhibitor, combotherapy

Supplementary Materiel & Methods

Immunofluorescence staining

mPDAC cells were seeded onto cover slips in 24-well plate (BD Falcon) to adhere overnight. The next day, cells were treated or not with the increasing concentrations of N6L. After 48h of treatment, cells were rinsed with cold PBS and fixed with 4% PFA for 10min/RT followed by permeabilization with 0.1% Triton X-100. The cells were subjected to immunofluorescence staining with phospho-RPS6 (CST) for 2h/RT then washed, incubated with Alexa 555-labeled anti-rabbit secondary antibody (BD Bioscience) for 1h/RT and then washed. Cell nuclei were counterstained with DAPI and cover slips were rinsed with distilled water and mounted using fluorescent mounting medium (Invitrogen). Cells were visualized under a confocal microscope (Zeiss LSM510).

RNAi transfection

A mixture of functional siRNAs (Eurogentec) specific for nucleolin was used as previously described [50]. siRNAs, siRNA #4 (UUCUUUGACAGGCUCUUCCUU) and siRNA #2 (UCCAAGGUAACUUUAUUUCUU), were reconstituted at a concentration of 100 nM and stored at −20 °C. As a siRNA control, we used stealth high GC siRNA (Invitrogen). Cells were transfected in a 6-well dish using siRNA at 2 nM final concentration. siRNAs were diluted in 200 µl of OptiMEM (Gibco) and plated in a well. 80 µl of INTERFERin (Polyplus) diluted 1:10 in RNase-free water were added. After 10min incubation, 2 ml of medium containing 3×10^5 cells were added. RNA extraction and protein analysis were performed 2 days after the initial transfection.

Live-cell imaging analysis

Cells were seeded in 96-well plates and allowed to adhere overnight or 2 days. Cells were treated with indicated concentrations of N6L and/or mTORi, for 48 or 72 hours. Cell growth was assessed by IncuCyte ZOOM® live-cell imaging to measure cell confluence after N6L and/or mTORi treatments. IncuCyte cell recognition software calculated values based on cellular size over time, cellular count per field of view or percentage of cell confluence over time (Essen Biosciences, Ann Arbor, MI).

Supplementary references

51. Li, H. *et al.* The Sequence Alignment/Map format and SAMtools. *Bioinformatics* **25**, 2078–2079 (2009).
52. Anders, S., Pyl, P. T. & Huber, W. HTSeq--a Python framework to work with high-throughput sequencing data. *Bioinformatics* **31**, 166–169 (2015).
53. Love, M. I., Huber, W. & Anders, S. Moderated estimation of fold change and dispersion for RNA-seq data with DESeq2. *Genome Biol.* **15**, 550 (2014).
54. Di Veroli, G. Y. *et al.* Combenefit: an interactive platform for the analysis and visualization of drug combinations. *Bioinformatics* **32**, 2866–2868 (2016).

Supplementary data legends

FIGURE S1: NCL targeting by N6L decreases mPDAC cell proliferation by inducing apoptosis.

mPDAC cells were seeded in 96-well plates and allowed to adhere overnight. Cells were treated with increasing concentrations of N6L. A- mPDAC cell growth was assessed by time-lapse Incucyte® over 120h. B- In parallel, mPDAC cell viability was determined by MTS assay periodically over 72h.

FIGURE S2: NCL targeting by N6L dysregulates mPDAC cell translation.

Bioinformatics analysis. **A-B,** Venn diagram showing the number of transcriptionally up-regulated, down-regulated or stable genes per Translation Index (TI) DOWN (C) or TI UP (D) category obtained in **fig.2. C-D**, GO terms for genes which are translationally down-regulated and transcriptionally up-regulated (n=340, green, C) or unchanged (n= 315, purple, D). **E,F-** GO terms for genes which are translationally up-regulated and transcriptionally down-regulated (n=131, red, E) or unchanged (n= 73, blue, F).

FIGURE S3: The impact of mTOR inhibitors treatment on mPDAC and PDX cell growth.

A,C- mPDAC cell lines were seeded in 96-well plates and allowed to adhere overnight. Cells were treated with 10 or 25nM of Rapamycin (RAPA, left), AZD2014 (AZD, middle) or INK128 (INK, right) over 72 hours. D-F, PDAC PDX (PAN014) cell lines were seeded in 96-well plates and allowed to adhere 2 days. Cells were treated with 1, 10 or 100nM of RAPA, AZD or INK over 96 hours. Cell growth was assessed by time-lapse InCucyte ZOOM®. The AUC (Area Under Curves) was calculated by InCucyte ZOOM® software. Growth for treated cells was normalized to untreated cells on the same plate.

FIGURE S4: **Synergistic effects of N6L/mTORi combination on MiaPaca cells**

A- MiaPaca cell lines were seeded in 96-well plates and allowed to adhere overnight. Cells were treated with concentrations of N6L alone at 10, 30 or 50µM (A, upper left), Rapamycin (RAPA) alone at 25 or 50nM (A, upper middle), INK128 (INK) alone at 10 or 25nM (A, upper right) or in combinations (A, lower) over 72 hours. Cell growth was assessed by time-lapse Incucyte®. The AUC (Area Under Curves) was calculated by Incucyte® software. Growth for treated cells was normalized to untreated cells on the same plate. B- Synergy/antagonism calculation. Additive and synergistic effects when using combinations of N6L with RAPA (B, left) or INK128 (B, right) were determined using Combenefit software. The software calculates a synergy score for each combination, where a positive score indicates synergy, a score of 0 is additive, and a negative score indicates antagonism. The Contour (B, upper), Surface (B, middle) and Matrix (B, lower) views from LOEWE model were selected as graphical outputs for the synergy distribution. C- Representative images of MiaPaca untreated (NT) or treated

with N6L alone or combined with RAPA (C, upper panels) or with INK128 (C, lower panels) at 48h after treatment. Scale bar, 50µm. D- Representative images of MiaPaca spheroids untreated (NT) or treated with N6L alone (upper panels) or combined with RAPA (D, middle panels) or with INK128 (D, lower panels) over 96h. E- MiaPaca spheroid area was calculated using ImageJ software and data were normalized to control for combinations of N6L and RAPA (upper graphs) or INK128 (lower graphs). (#) if comparing each treatment to NT, (*) if comparing combination to single treatment. *P<0.05; **P<0.01; ***P<0.001; ****P<0.0001 and n.s. for not statistically significant, as determined by *Student t-test* on final data point. F- N6L combined to mTORi treatment inactivate mTOR pathway by decreasing RPS6 and 4EBP1 phosphorylation. mTOR pathway inhibition was analyzed using western-blotting in MiaPaca cells, non-treated (NT) or treated with N6L, RAPA, INK128 alone or in combination (N6L+RAPA, N6L+INK).

FIGURE S5: **Synergistic effects of N6L/mTORi combination on Panc cells**

A- Panc cell lines were seeded in 96-well plates and allowed to adhere overnight. Cells were treated with concentrations of N6L alone at 10, 30 or 50µM (A, upper left), Rapamycin (RAPA) alone at 25 or 50nM (A, upper middle), INK128 (INK) alone at 10 or 25nM (A, upper right) or in combinations (A, lower) over 72 hours. Cell growth was assessed by time-lapse Incucyte®. The AUC (Area Under Curves) was calculated by Incucyte® software. Growth for treated cells was normalized to untreated cells on the same plate. B- Synergy/antagonism calculation. Additive and synergistic effects when using combinations of N6L with RAPA (B, left) or INK128 (B, right) were determined using Combenefit software. The software calculates a synergy score for each combination, where a positive score indicates synergy, a score of 0 is additive, and a negative score indicates antagonism. The Contour (B, upper), Surface (B, middle) and Matrix (B, lower) views from LOEWE model were selected as graphical outputs for the synergy distribution. C- Representative images of Panc untreated (NT) or treated with N6L alone or combined with RAPA (C, upper panels) or with INK128 (C, lower panels) at 48h after treatment. Scale bar, 50µm. D- Representative images of Panc spheroids untreated (NT) or treated with N6L alone (upper panels) or combined with RAPA (D, middle panels) or with INK128 (D, lower panels) over 96h. E- Panc spheroid area was calculated using ImageJ software and data were normalized to control for combinations of N6L and RAPA (upper graphs) or INK128 (lower graphs). (#) if comparing each treatment to NT, (*) if comparing combination to single treatment. *P<0.05; **P<0.01; ***P<0.001; ****P<0.0001 and n.s. for not statistically significant, as determined by *Student t-test* on final data point. F- N6L combined to mTORi treatment inactivate mTOR pathway by decreasing RPS6 and 4EBP1 phosphorylation. mTOR

pathway inhibition was analyzed using western-blotting in Panc cells, non-treated (NT) or treated with N6L, RAPA, INK128 alone or in combination (N6L+RAPA, N6L+INK).

FIGURE S6: Originals western blot images.
