# Supplementary material for: Nucleolin Aptamer N6L Reprograms the Translational Machinery and Acts Synergistically with mTORi to Inhibit Pancreatic Cancer Proliferation
Source: Cancers (Basel). 2021 Oct 1;13(19):4957. doi: 10.3390/cancers13194957 (PMC8508287; doi:10.3390/cancers13194957)
Supplement: Supplementary file 1 [file cancers-13-04957-s001.zip › cancers-1318085-SI/Chalabi_Sup Table 2_14062021.pdf]

Supplementary table S2

Primary antibodies	Dilution	References
**Nucleolin**	1:1000	#22758; Abcam
**Puromycin (12D10)**	1:2000	#MABE343; EMD Millipore
**Actin**	1:5000	#A2228; Sigma-Aldrich
**Histone H3**	1:3000	#1791; Abcam
**RPS6**	1:1000	#2317; Cell Signaling Technology
**Phospho-RPS6**	1:1000	#2211; Cell Signaling Technology
**mTOR**	1:1000	#2972; Cell Signaling Technology
**Phospho-mTOR**	1:1000	#2971; Cell Signaling Technology
**Pan-AKT**	1:1000	#9272; Cell Signaling Technology
**Phospho-AKT**	1:500	#9271; Cell Signaling Technology
**4EBP1**	1:1000	#9644; Cell Signaling Technology
**Phospho-4EBP1**	1:1000	#2855; Cell Signaling Technology
**LARP1**	1:1000	#14763; Cell Signaling Technology
**EiF4E**	1:1000	#9742; Cell Signaling Technology
**EiF3a**	1:1000	#NBP1-18891,NovusBio
**EiF3c**	1:1000	#A300-377A ; Bethyl
**EiF3e**	1:1000	#ab36766 ; Abcam
**EiF3h**	1:1000	#A301-754A ; Bethyl
**EiF3k**	1:1000	#NB100-93304. NovusBio
**RPS3**	1:500	#A303-840A ; Bethyl
**RPL36A**	1:500	#ab241584 ; Abcam
**Secondary antibodies**	**Dilution**	**References**
**Anti-rabbit IgG**	1:10000	#7074; Cell Signaling Technology
**Anti-mouse IgG**	1:20000	#7076; Cell Signaling Technology
