# Supplementary material for: Nucleolin Aptamer N6L Reprograms the Translational Machinery and Acts Synergistically with mTORi to Inhibit Pancreatic Cancer Proliferation
Source: Cancers (Basel). 2021 Oct 1;13(19):4957. doi: 10.3390/cancers13194957 (PMC8508287; doi:10.3390/cancers13194957)

Supplementary figure S1

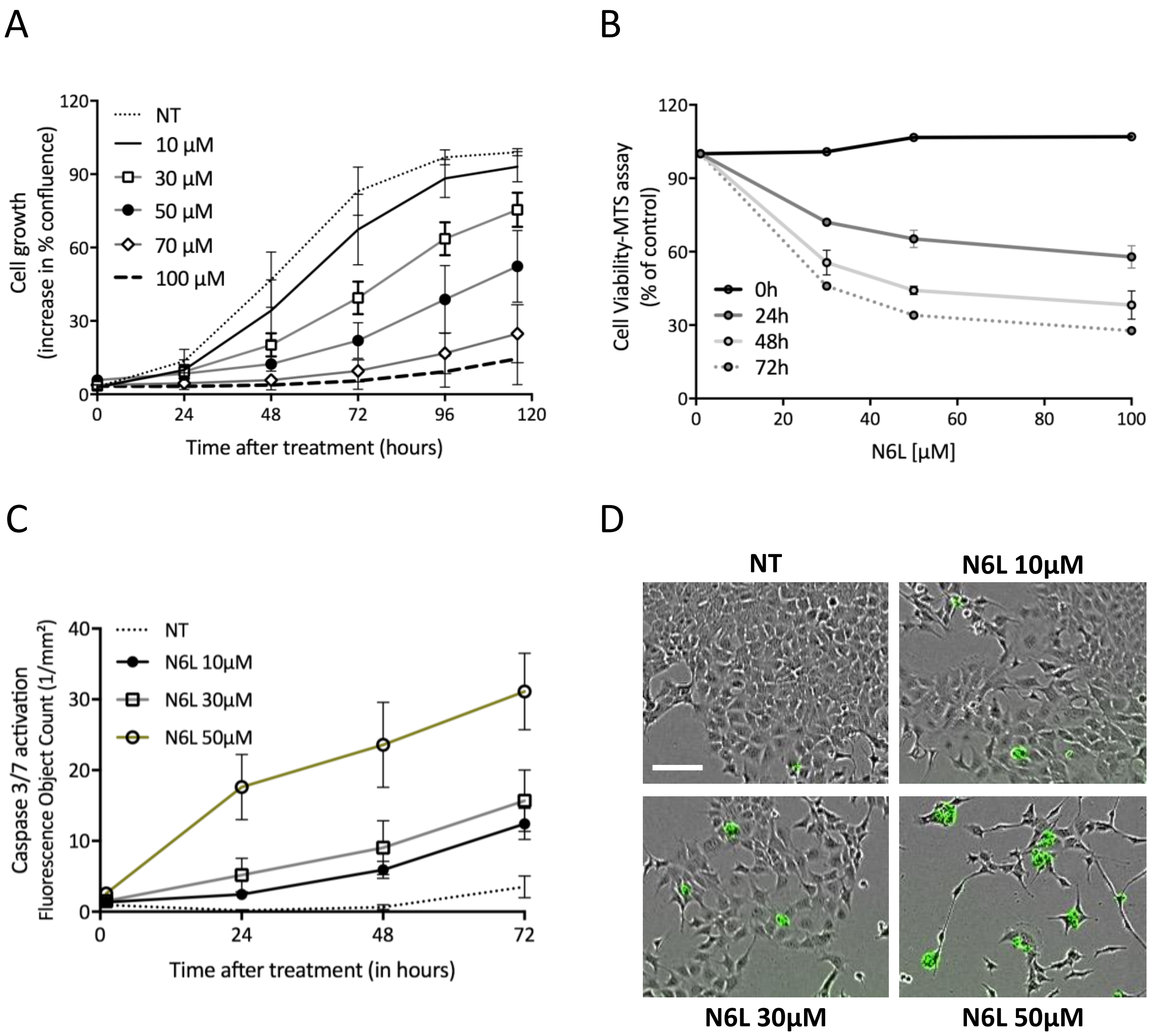

Supplementary data S2

A

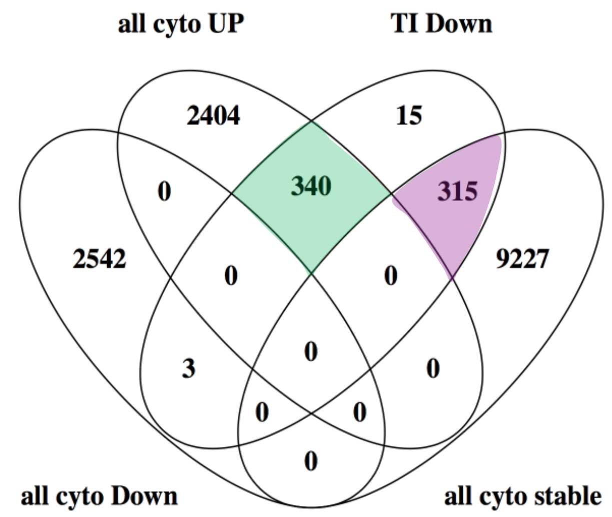


B

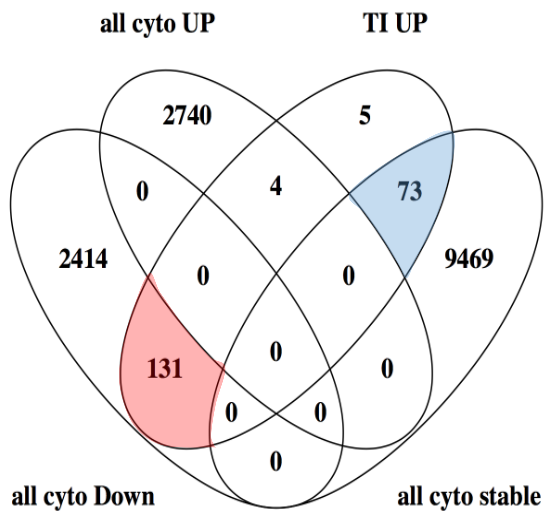


C

**TI Down/Cyto UP (n=340)**

| Index | Name | P-value | Adjusted p-value | Odds Ratio | Combined score |
|---|---|---|---|---|---|
| 1 | PIP biosynthesis at the early endosome membrane | 0.0009575 | 0.2410 | 14.79 | 102.83 |
| 2 | PIP biosynthesis at the plasma membrane | 0.0001484 | 0.1120 | 9.71 | 85.62 |
| 3 | PIP biosynthesis at the Golgi membrane | 0.002174 | 0.2984 | 11.31 | 69.36 |
| 4 | Phosphatidylinositol metabolism | 0.0001225 | 0.1850 | 7.69 | 69.29 |
| 5 | PPAR-gamma coactivator role in obesity and thermogenesis | 0.008123 | 0.4906 | 14.25 | 68.56 |

E

**TI UP/Cyto Down (n=131)**

| Index | Name | P-value | Adjusted p-value | Odds Ratio | Combined score |
|---|---|---|---|---|---|
| 1 | Translation | 1.882e-11 | 1.421e-8 | 13.25 | 327.10 |
| 2 | Cytoplasmic ribosomal proteins | 2.143e-9 | 0.000001079 | 14.25 | 284.35 |
| 3 | Mitochondrial protein import | 0.000001070 | 0.0002020 | 17.75 | 244.05 |
| 4 | Influenza viral RNA transcription and replication | 8.943e-9 | 0.000003376 | 12.31 | 228.09 |
| 5 | Activation of mRNA upon binding of the cap-binding complex and eIFs, and subsequent binding to 43S | 0.000001857 | 0.0003116 | 16.19 | 213.71 |

D

**TI Down/Cyto stable (n=315)**

| Index | Name | P-value | Adjusted p-value | Odds Ratio | Combined score |
|---|---|---|---|---|---|
| 1 | mitotic sister chromatid cohesion (GO:0007064) | 0.000002477 | 0.0007435 | 21.16 | 273.20 |
| 2 | regulation of cell cycle G2/M phase transition (GO:1902749) | 5.105e-11 | 1.303e-7 | 10.58 | 250.77 |
| 3 | positive regulation of metaphase/anaphase transition of cell cycle (GO:1902101) | 0.0001292 | 0.01534 | 27.21 | 243.64 |
| 4 | positive regulation of mitotic sister chromatid separation (GO:1901970) | 0.0001292 | 0.01499 | 27.21 | 243.64 |
| 5 | mitotic sister chromatid segregation (GO:0000070) | 4.827e-10 | 4.106e-7 | 10.07 | 215.93 |

F

**TI UP/Cyto stable (n=73)**

| Index | Name | P-value | Adjusted p-value | Odds Ratio | Combined score |
|---|---|---|---|---|---|
| 1 | regulation of cholesterol storage (GO:0010885) | 0.0008470 | 1.000 | 45.66 | 323.01 |
| 2 | cellular response to nitrogen starvation (GO:0006995) | 0.0009986 | 1.000 | 42.15 | 291.22 |
| 3 | cellular response to nitrogen levels (GO:0043562) | 0.0009986 | 1.000 | 42.15 | 291.22 |
| 4 | positive regulation of lipid storage (GO:0010884) | 0.002158 | 1.000 | 28.84 | 177.03 |
| 5 | peroxisomal membrane transport (GO:0015919) | 0.02170 | 1.000 | 45.66 | 174.90 |

Supplementary data S3

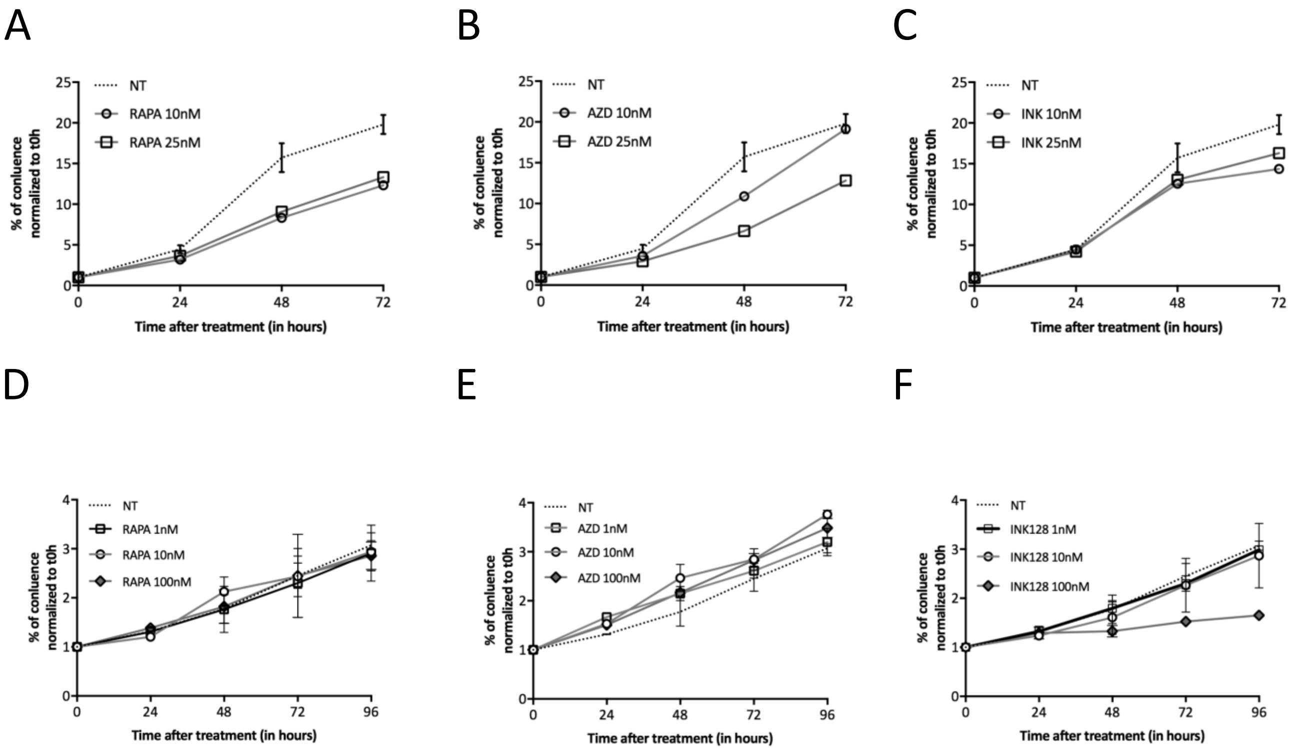

Supplementary data S4

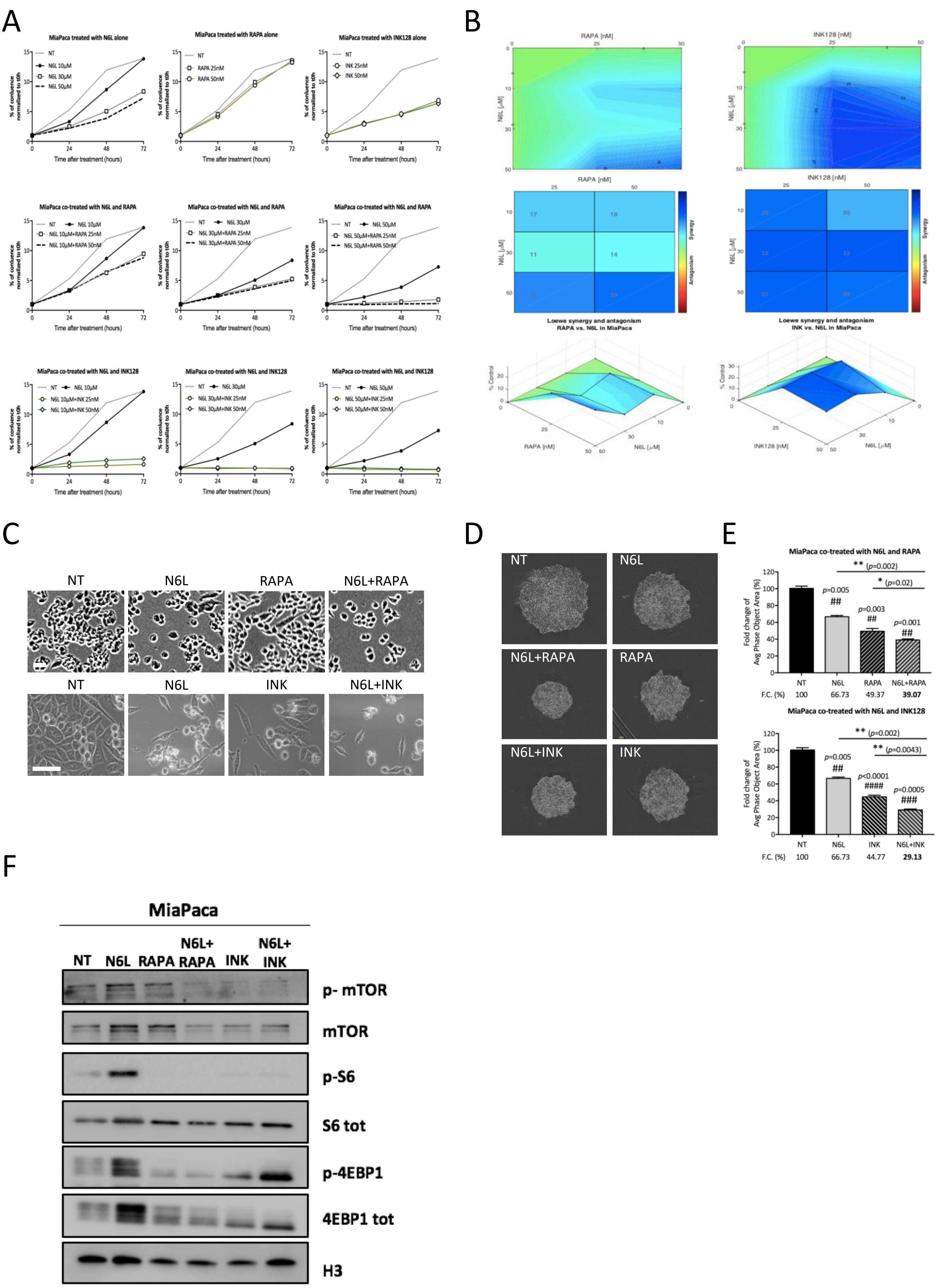

Supplementary data S5

A

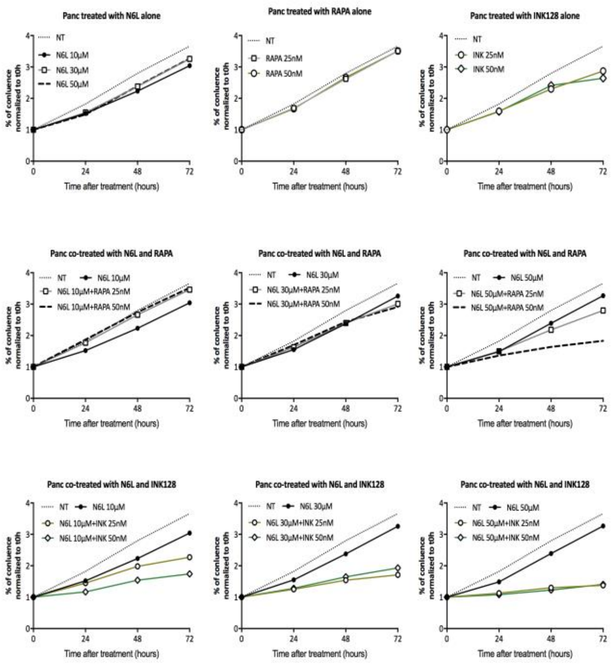

B

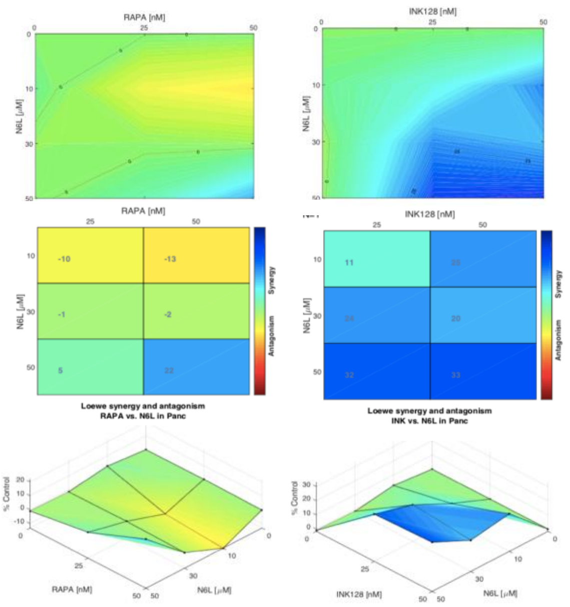

C

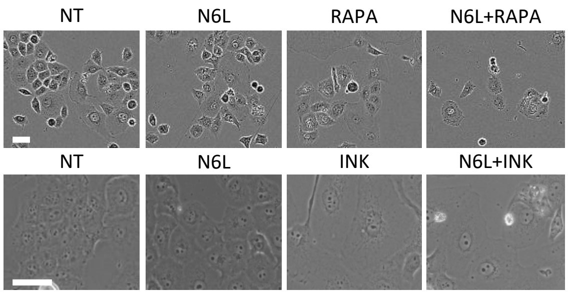

D

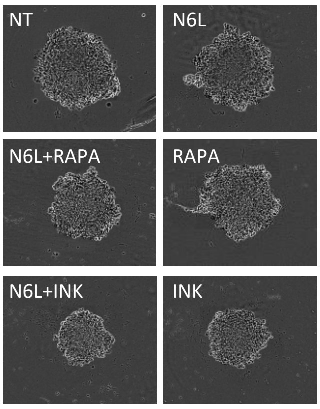

E

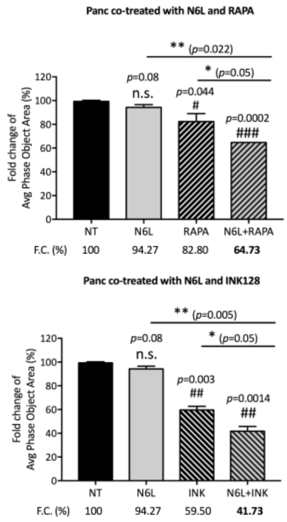

F

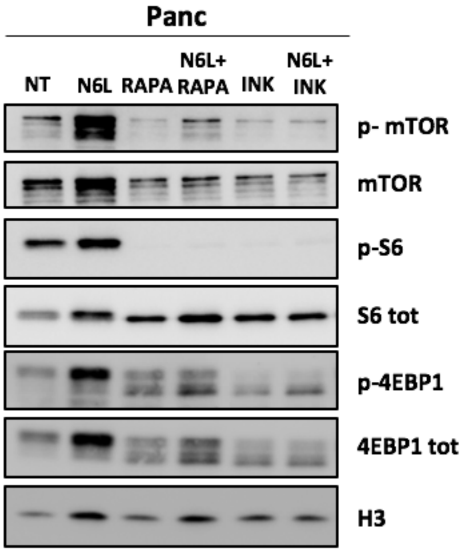

Supplementary Figure S6

A-uncropped WB for Fig 1D

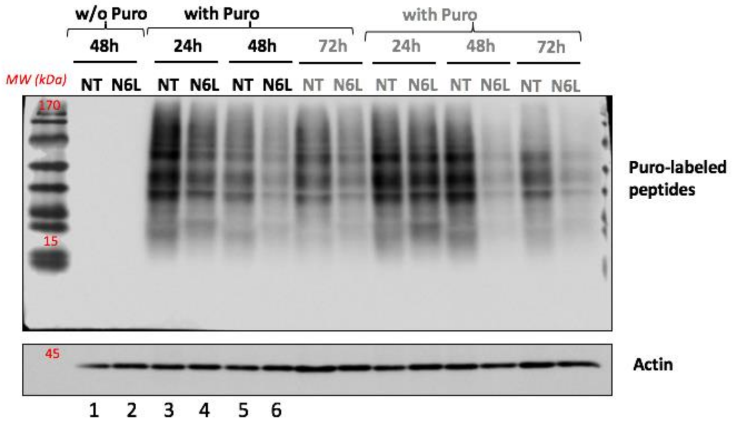


B- uncropped WB for Fig 3B-D

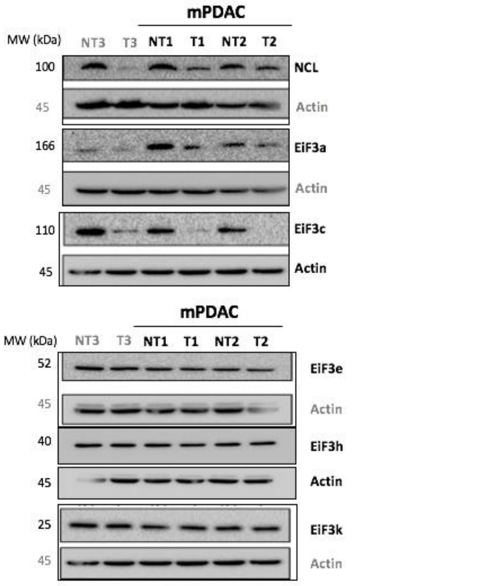


C- uncropped WB for Fig 4D

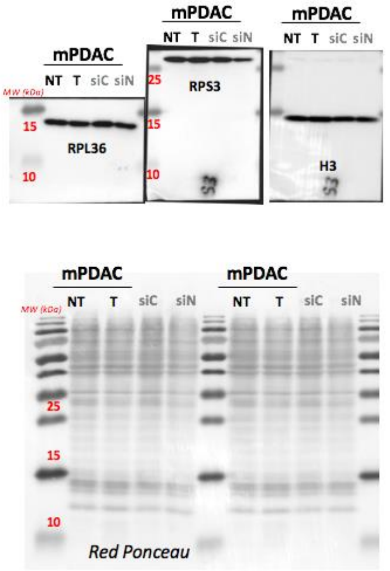


D- uncropped WB for Fig 5D

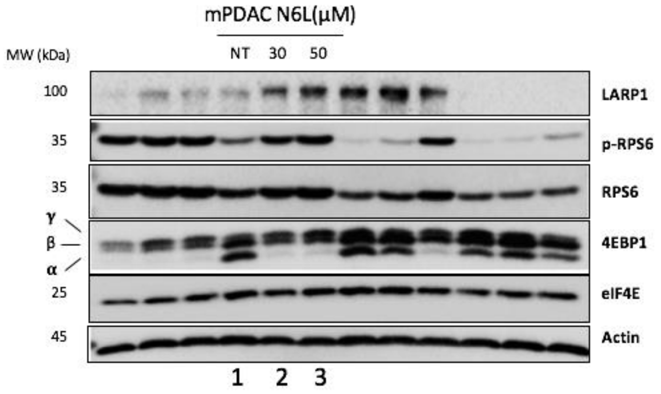


E-uncropped WB for Fig 7A

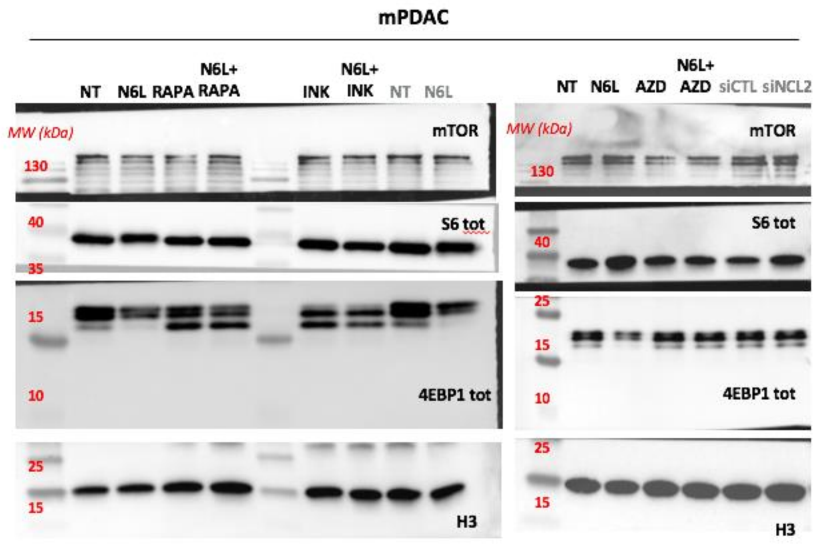

Supplementary Figure S6

F-uncropped WB for Fig 7B

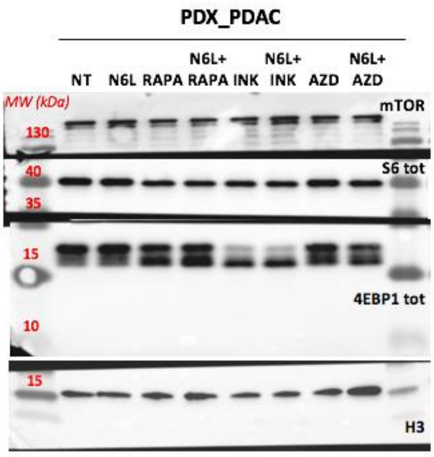


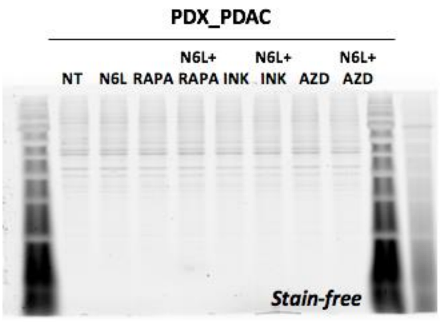


G-uncropped WB for Fig S4F & S5F

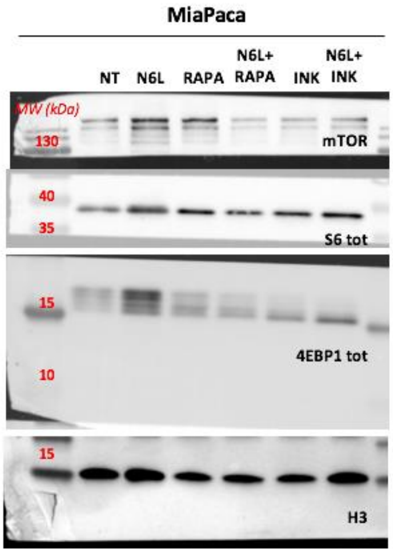


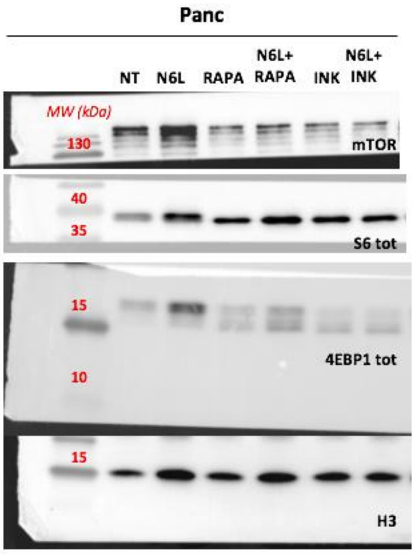

Supplement: Supplementary file 1 [file cancers-13-04957-s001.zip › cancers-1318085-SI/ms Transla_mTORi_Suppdata_30092021.pdf]
